# Persistent impairments in muscle function and symptom burden in post-COVID syndrome: a prospective longitudinal study

**DOI:** 10.1186/s12967-026-08579-z

**Published:** 2026-07-07

**Authors:** Michael Wunderle, Andrea Ribeiro, Isabelle Lethen, Christoph Schmaderer, Timon Wallraven

**Affiliations:** 1 Department of Nephrology, TUM School of Medicine and Health, TUM University Hospital, Technical University of Munich, Munich, Germany; 2 German Centre for Infection Research (DZIF), Munich Partner Site, Munich, Germany; 3 VADYS-ME Research Program, Site Lead, TUM University Hospital, Munich, Germany

**Keywords:** Post-COVID syndrome, Long COVID, Handgrip strength, Muscle impairment, Fatigability, Neurofilament light chain, GFAP, Longitudinal study

## Abstract

**Background:**

Post-COVID syndrome (PCS) is characterized by persistent heterogeneous symptoms after SARS-CoV-2 infection, yet objective biomarkers for symptom severity and longitudinal disease trajectories remain limited. We aimed to characterize muscle function over time in PCS and examine its relationship with symptom burden and neuroaxonal injury markers.

**Methods:**

In this prospective observational study, patients fulfilling WHO criteria for PCS underwent standardized assessments at baseline (BL) and six-month follow-up (FU). Muscle function was assessed using a multidimensional handgrip strength (HGS) protocol capturing mean force (Fmean), fatigability (fatigue ratio), and recovery capacity alongside clinical symptom measures. Analyses included longitudinal assessment within the PCS cohort, comparisons with COVID-19 recovered controls, analysis of symptom persistence, and matched cohort analyses including HGS measures and circulating neurofilament light chain (NfL) and glial fibrillary acidic protein (GFAP).

**Results:**

Among 204 participants (102 PCS, 102 recovered controls) PCS patients showed lower Fmean and higher fatigability at BL and FU (both *p* < 0.01). Muscle function parameters remained largely stable over six months, with only modest improvement in recovery (*p* = 0.015). HGS parameters correlated with symptom burden (ρ ≈ -0.28 to -0.33), and BL impairments were associated with worse fatigue and depressive symptoms at FU (e.g., Fmean–PHQ-9: β = -0.11, *p* = 0.022; fatigue ratio–FSS: β = 1.66, *p* = 0.008). Exploratory analyses suggested slightly higher NfL and GFAP levels in PCS in some models, without consistent associations with muscle function or symptoms.

**Conclusions:**

HGS is persistently reduced in PCS over at least six months and aligns with symptom burden. Multidimensional HGS assessment may provide a practical objective marker of functional impairment and symptom persistence in PCS. Neuroaxonal injury markers showed modest elevations in PCS in some analyses but appeared unrelated to muscle performance, suggesting partially distinct mechanisms.

**Trial registration:**

ClinicalTrials.gov, NCT05635552. Registered 1 December 2022 - Retrospectively registered, https://clinicaltrials.gov/study/NCT05635552?term=NCT05635552&rank=1&tab=study.

**Supplementary Information:**

The online version contains supplementary material available at 10.1186/s12967-026-08579-z.

## Introduction

The COVID-19 pandemic has left an enduring legacy beyond its acute phase, with millions of individuals worldwide experiencing persistent symptoms months after initial infection, a condition recognized as post-COVID syndrome (PCS) or long COVID [1, 2]. The World Health Organization defines PCS as symptoms persisting for at least three months after SARS-CoV-2 infection, lasting at least two months, and not explained by alternative diagnoses [3]. PCS represents a major public health challenge affecting millions of COVID-19 survivors [4, 5]. Given its multisystem nature [6, 7], understanding the natural history and persistence of objective physical impairments has become a critical research priority.

Among the most debilitating manifestations of PCS are physical impairments, particularly muscle weakness, reduced exercise capacity, and persistent fatigue [8–10]. Handgrip strength (HGS) is a validated and widely used objective marker of overall muscle function and physical performance and has been shown to be reduced in PCS compared with recovered controls [11–13]. Beyond maximal force, repeated HGS assessments allow quantification of fatigability and recovery, providing a multidimensional characterization of muscle performance relevant to PCS-related fatigue and exercise intolerance [12, 14]. Lower HGS has been associated with worse functional outcomes and quality of life in post-COVID populations [11, 15, 16].

Despite growing recognition of reduced handgrip strength in PCS, critical gaps remain in the literature, particularly regarding the distinction between post-acute COVID-19 patients and well-defined chronic PCS cohorts. Many existing studies have focused on the early recovery phase (within the first three months post-infection), often including heterogeneous populations without rigorous application of diagnostic criteria [16–21]. Systematic reviews have highlighted that muscle function impairments can persist up to 12 months in PCS patients, yet longitudinal studies with objective measurements in well-characterized cohorts remain scarce [6, 22]. Cross-sectional investigations, while informative, cannot address fundamental questions about the temporal stability of muscle impairments or identify predictive factors for symptom persistence [18, 23].

Understanding the biological mechanisms underlying reduced muscle performance in PCS requires integration of multiple assessment modalities. Impairments in muscle function may reflect broader neuromuscular or neurovascular involvement, including small-fiber-neuropathy [24, 25], endothelial dysfunction and dysfunctional neurovascular coupling [26]. Emerging evidence suggests that PCS may be associated with elevated neuroaxonal injury biomarkers, including neurofilament light chain (NfL, marker of axonal damage) and glial fibrillary acidic protein (GFAP, marker if astroglial activation and inflammation), while evidence for overt neurodegeneration remains inconsistent [27–29]. Given the overlap between neurological and musculoskeletal manifestations in PCS, particularly the co-occurrence of cognitive impairment, fatigue, and muscle weakness, there is a compelling rationale to explore potential associations between objective muscle function measures and neuroaxonal injury markers.

Moreover, few studies have employed comprehensive HGS protocols that capture multiple dimensions of muscle function impairments (strength, fatigability, and recovery) or have integrated such objective physical measurements with patient-reported outcome measures (PROMs) and biomarkers of neuroaxonal injury [27, 30, 31]. The lack of prospective longitudinal data in rigorously defined PCS cohorts represents a significant knowledge gap that limits our understanding of disease trajectory and prognosis.

To address this gap, the present study provides a prospective, longitudinal assessment of muscle function in a well-defined PCS cohort followed over six months. Using a multidimensional HGS protocol, objective muscle function was assessed alongside validated PROMs and neuroaxonal injury biomarkers. Comparisons with age- and sex-matched COVID-19 recovered controls enabled isolation of PCS-specific impairments beyond general post-infectious effects.

The primary aims were to characterize HGS parameters at baseline (BL) and six months follow-up (FU), compare muscle function between PCS patients and matched controls, assess longitudinal changes in objective and PROMs, and examine associations with neuroaxonal injury biomarkers and symptom severity. Together, these analyses aim to clarify the persistence of objective muscle impairments in chronic PCS and identify prognostic markers of long-term symptom burden.

## Methods

### Study design and cohort

This study was conducted as part of the “All Eyes on PCS” project, a prospective, observational, single-center study at the Technical University of Munich (Departments of Nephrology, Neurology, and Ophthalmology). The overarching aim of the project was to investigate retinal microvascular alterations and to provide detailed clinical characterization of patients with post-COVID syndrome (PCS). Recruitment procedures and BL assessments have been described previously [32].

Eligible participants were adults with confirmed prior SARS-CoV-2 infection (polymerase chain reaction or serological evidence ≥ 3 months before inclusion) and persistent PCS-related symptoms for at least two months without an alternative medical explanation. Exclusion criteria comprised lack of informed consent, age < 18 years, pregnancy, active malignancy, conditions markedly limiting life expectancy, rheumatologic autoimmune diseases, and relevant ocular or neurological disorders (e.g., cataract, glaucoma, epilepsy), reflecting the requirements of the overarching retinal vascular imaging study protocol [32].

Between October 2022 and September 2023, 105 individuals with suspected PCS were enrolled, including 76 recruited via social media outreach and 29 from the PCS outpatient clinic. Three participants were excluded from the final analysis: one due to missing confirmation of SARS-CoV-2 infection, one because no temporal relationship between infection and symptom onset could be established, and one whose PCS-typical symptoms had resolved prior to inclusion. We recruited a control group of individuals who had fully recovered from a previous SARS-CoV-2 infection without persistent symptoms between December 2022 and January 2025.

We invited PCS participants for a standardized follow-up visit six months after BL assessment. During this FU visit, patient-reported outcome measures (PROMs), laboratory parameters, and HGS testing were repeated using the same protocols as at BL. FU assessments were used to evaluate longitudinal changes within the PCS cohort and to compare FU values with BL measurements from recovered controls in selected analyses.

All examinations were performed by trained investigators familiar with the study procedures but not involved in hypothesis formulation or statistical analysis. The study protocol was approved by the Ethics Committee of the Technical University of Munich, School of Medicine (Klinikum rechts der Isar; approval number 2022-317-S-SR) and prospectively registered in November 2022 at ClinicalTrials.gov (identifier: NCT05635552). Registration occurred after study initiation because of delays in the registration process. The study protocol remained unchanged after registration. The study was conducted in accordance with the Declaration of Helsinki and institutional ethical standards, and all participants provided written informed consent prior to inclusion.

### Sample size

Sample size calculations were originally performed for retinal vessel analyses, a primary endpoint of the “All Eyes on PCS” study. Based on published normative data and expected group differences, we defined a target of approximately 100 participants per group to ensure adequate statistical power while accounting for potential dropouts. The analyses presented here represent a predefined substudy within this larger framework.

### Assessment of outcome measures

#### Neuroaxonal injury markers (NfL and GFAP)

Venous blood samples were obtained by trained study personnel during morning hours and processed according to standardized operating procedures. Serum was aliquoted immediately after centrifugation and stored at -80 °C until analysis. All samples underwent a single freeze-thaw cycle.

Serum concentrations of neurofilament light chain (NfL) and glial fibrillary acidic protein (GFAP) were quantified using Single Molecule Array (Simoa^®^) technology on the HD-X Analyzer (Quanterix, Billerica, MA, USA) with the commercially available Neurology 2-Plex B Advantage Kit, following the manufacturer’s instructions. Lower limits of quantification were 0.8 pg/mL for NfL and 16.6 pg/mL for GFAP. Samples below the lower limit of quantification or with technical assay artifacts were excluded. Laboratory personnel were blinded to all clinical and outcome data.

#### Handgrip strength assessment

We measured HGS of the dominant hand using a calibrated digital dynamometer (Jamar^®^, Patterson Medical) under standardized conditions according to established HGS testing recommendations [33]: Participants were seated upright with the elbow flexed at 90° and the wrist in a neutral position. After familiarization, we assessed HGS in two standardized sessions separated by a 60-minute rest period. Each session consisted of ten maximal voluntary contractions with brief rest intervals of approximately 3 s.

The following parameters were derived as previously described [14] for each session: mean grip force (Fmean; $\:{\Sigma\:}$10 measurements/10), Fatigue Ratio (maximum force/Fmean), and muscle Recovery (Fmean of session 2/ Fmean of session 1). This multidimensional protocol has previously been applied to characterize fatigability and recovery deficits in ME/CFS and PCS-related conditions [14].

Normative percentiles were taken from a large, published reference dataset in which handgrip strength values were harmonized to three trials using multivariable modelling [34]. In our cohort, handgrip strength was calculated as the mean of 10 repeated measurements; therefore, comparisons with normative percentiles should be interpreted as an approximation.

#### Patient-reported outcome measures (PROMs)

We assessed symptom burden and functional impairment using validated PROMs. PCS-related symptom severity was quantified using the COVID-19 Yorkshire Rehabilitation Scale (C19-YRS) [35] and the PCS Severity Score [36]. Fatigue was evaluated with the Fatigue Severity Scale (FSS) [37]. Symptoms of depression and anxiety were assessed using the 9-item Patient Health Questionnaire (PHQ-9) [38] and the Generalized Anxiety Disorder Scale (GAD-7) [39], respectively. Higher scores indicate greater symptom severity for all instruments. All PROMs were assessed at BL and repeated at the FU visit in the PCS cohort.

### Statistical analysis

All analyses were performed in R (version 2026.01.0 + 392 [2026.01.0 + 392]). Continuous variables are reported as mean ± standard deviation (SD) or median (interquartile range [IQR]), and categorical variables as n (%). Group comparisons used ANOVA or Kruskal–Wallis tests for continuous variables and χ² or Fisher’s exact tests for categorical variables, as appropriate. Analyses were conducted as complete-case for the variables included in each model. Implausible recovery values (> 1.25) were set to missing.

Within the PCS cohort, longitudinal BL-FU changes were analyzed using paired t-tests or Wilcoxon signed-rank tests depending on data distribution. Longitudinal time effects on muscle function parameters were additionally assessed using linear mixed-effects models with fixed effects for time, age, and sex and a random intercept for participant ID. Missing FU data were evaluated using Little’s MCAR test; because missingness was not completely at random, mixed-effects models were used under a missing-at-random assumption.

Spearman correlations were used to assess associations between muscle function parameters and PROMs at BL and FU, as well as associations between longitudinal changes (Δ = FU − BL). Associations between BL function and FU outcomes were assessed using univariable and multivariable linear regression models examining whether BL muscle function was associated with FU muscle function and PROMs. Multivariable models adjusted for age, sex, and the corresponding BL PROM. For cross-sectional group comparisons between PCS patients and healthy controls, multivariable linear regression models were applied, including group (PCS vs. control), age, and sex as covariates. Interaction analyses included ME/CFS status as an effect modifier. Heteroskedasticity-consistent HC3 robust standard errors were used where appropriate. Multiple testing was controlled using the Benjamini-Hochberg false discovery rate (FDR) method.

Propensity score matching was performed using the MatchIt package in R according to established recommendations for observational studies [40]. Propensity scores were estimated using logistic regression including age and sex as covariates. Nearest-neighbor matching with replacement was performed targeting the average treatment effect on the treated (ATT) using a 2:1 control-to-PCS ratio and a caliper of 0.5. Covariate balance was assessed using standardized mean differences (SMDs). Matched analyses incorporated ATT weighting and used weighted linear models with HC3 robust standard errors [41]. NfL and GFAP were analyzed on the log scale and additionally adjusted for eGFR as an established confounder. All tests were two-sided with α = 0.05.

## Results

### Baseline characteristics of the study cohort

A total of 204 participants were included: 102 PCS patients and 102 COVID-19 recovered controls (Table 1). PCS patients were significantly older than recovered individuals (median 41.5 [32.0–52.0] vs. 30.0 [25.0–37.8] years, *p* < 0.001) but showed comparable sex distribution (75.5% vs. 71.6% female, *p* = 0.63). No differences were observed in BMI, obesity, or smoking status, whereas hypertension (*p* = 0.019) and hypercholesterolemia (*p* < 0.001) were more frequent in PCS patients. PCS participants reported more severe acute COVID-19 courses (*p* < 0.001). Time since infection was slightly shorter in PCS patients (462 ± 268 days vs. 555 ± 359 days, *p* = 0.041). Vaccination status differed between groups, with PCS patients being less frequently vaccinated and more often infected before vaccination (both *p* < 0.001). ME/CFS was present in 61.4% of PCS patients. At BL, PCS patients showed markedly higher fatigue severity (FSS median 6.1 [5.1–6.7] vs. 2.2 [1.5–3.1], *p* < 0.001) and significantly lower muscle strength (Fmean 20.1 [14.6–27.0] vs. 28.8 [23.6–34.6] kg, *p* < 0.001). Fatigue ratio was slightly higher (*p* = 0.028), and recovery was significantly lower (*p* < 0.001) in the PCS group.

Of 102 PCS participants, 87 (85%) completed the FU. A Little’s MCAR test indicated that missingness was not completely at random. Baseline comparisons between participants with and without FU showed no statistically significant differences in Biomarkers or PROMs (all *p* > 0.05; Supplementary Table 1), although a non-significant trend toward lower baseline symptom burden was observed in those lost to FU.

**Table 1:** BL characteristics of patients with PCS and COVID-19 recovered controls. Values are expressed as mean ± standard deviation (SD), median (interquartile range, IQR), number (percentage), or as the most frequent category (n %) for multi-category variables, as appropriate. P-values are derived from one-way ANOVA for normally distributed continuous variables, Kruskal-Wallis tests for non-parametric continuous variables, and χ² or Fisher’s exact tests for categorical variables

Variable	COVID-19 recovered (*n* = 102)	PCS patients (*n* = 102)	*p*-value
Age, years	30.0 (25.0–37.8)	41.5 (32.0–52.0)	< 0.001
Female sex, n (%)	73 (71.6%)	77 (75.5%)	0.63
Body mass index, kg/m²	22.8 (21.1–24.7)	23.7 (21.0–27.0)	0.15
Obesity, n (%)	8 (7.8%)	16 (15.7%)	0.13
Nicotine abuse, n (%)	14 (13.7%)	10 (9.8%)	0.52
Hypertension, n (%)	5 (4.9%)	16 (15.7%)	0.019
Diabetes mellitus type II, n (%)	0 (0.0%)	1 (1.0%)	1.0
Hypercholesterolemia, n (%)	21 (22.1%)	46 (46.5%)	< 0.001
Severity of acute infection, n (%)			< 0.001
** Low (0–2)**	91 (89.2%)	62 (60.8%)	
** Moderate (3–5)**	10 (9.8%)	39 (38.2%)	
** High (> 5)**	1 (1.0%)	1 (1.0%)	
SARS-CoV-2 variant, n (%)			0.002
** unknown**	80 (79.2%)	63 (61.8%)	
** alpha**	1 (1.0%)	7 (6.9%)	
** delta**	2 (2.0%)	13 (12.7%)	
** omikron**	18 (17.8%)	19 (18.6%)	
Number of SARS-CoV-2 infections, n (%)			0.11
** 1**	62 (60.8%)	71 (69.6%)	
** 2**	31 (30.4%)	29 (28.4%)	
** ≥3**	9 (8.8%)	2 (2.0%)	
Time since infection, days	555.0 ± 358.9	462.2 ± 267.6	0.041
Number of vaccinations, n (%)			< 0.001
** 0**	0 (0.0%)	5 (4.9%)	
** 1**	1 (1.0%)	4 (3.9%)	
** 2**	8 (7.9%)	27 (26.5%)	
** ≥3**	92 (90.2%)	66 (64.7%)	
Vaccinated before infection, n (%)	6 (6.0%)	27 (27.6%)	< 0.001
ME/CFS diagnosis, n (%)	0 (0.0%)	62 (61.4%)	< 0.001
C19-YRS	–	32.7 ± 14.1	–
GAD-7 score	–	5.0 (2.0–9.0)	–
PHQ-9 score	–	10.4 ± 4.3	–
Fatigue Severity Scale	2.2 (1.5–3.1)	6.1 (5.1–6.7)	< 0.001
PCS Severity Score	–	36.9 ± 10.6	–
Fmean (kg)	28.6 (22.9–34.2)	20.1 (14.6–27.0)	< 0.001
Fatigue ratio	1.2 (1.1–1.2)	1.2 (1.1–1.3)	0.035
Recovery	1.0 (0.9–1.1)	1.0 (0.8–1.0)	< 0.001

### Matched cohort analysis (Primary analysis)

Given the substantial baseline differences, particularly in age, between PCS patients and recovered controls, a propensity score-matched analysis was considered the primary approach for between-group comparisons. Matched analyses including neuroaxonal injury markers were conducted to explore potential biological correlates of impaired muscle function.

#### Matching procedure and balance

A total of 102 patients with PCS were matched to 102 COVID-19 recovered controls using 2:1 nearest-neighbor propensity score matching with replacement, targeting the average treatment effect on the treated. The PCS cohort remained unchanged in the matched analyses. Although 63 unique recovered individuals contributed to the matched sample, weighting resulted in an effective control group representing 102 recovered controls (effective sample size 34.7). Matching was performed on age and sex to account for BL differences between groups (Table 1). Covariate balance substantially improved after matching, with standardized mean differences (SMDs) for all matching variables below 0.1, indicating adequate balance (Supplementary Fig. 1). Table 2 shows the weighted BL demographics of matched cohort.

**Table 2:** BL characteristics of patients with PCS and COVID-19 recovered controls in the age- and sex-matched cohort. Values are mean ± SD or median (IQR), as appropriate

Variable	COVID recovered (*n* = 63 unique; weighted *n* ≈ 102; ESS = 34.7)	PCS *n* = 102	SMD
**Demographics and comorbidities**			
Age, years	41.9 ± 12.1	41.9 ± 11.6	0.001
Female sex, n (%)	48 (76.0%)	77 (75.5%)	0.011
BMI, kg/m²	24.1 ± 4.1	24.5 ± 4.8	0.096
Obesity, n (%)	9 (13.7%)	16 (15.7%)	0.055
Nicotine abuse, n (%)	7 (11.8%)	10 (9.8%)	0.063
Hypertension, n (%)	10 (15.7%)	16 (15.7%)	0.000
Diabetes mellitus type II, n (%)	0 (0.0%)	1 (1.0%)	0.141
Hypercholesterolemia, n (%)	25 (41.2%)	46 (46.5%)	0.106
**COVID-19-related characteristics**			
**Severity of acute infection**			
** low (0–2)**, n (%)	56 (90.3%)	62 (60.8%)	0.713
** moderate (3–5)**, n (%)	6 (9.7%)	40 (39.2%)	0.713
** high (> 5)**, n (%)	0 (0.0%)	2 (2.0%)	
**SARS-CoV-2 variant**			
unknown, n (%)	49 (78.4%)	63 (61.8%)	0.432
Alpha, n (%)	2 (2.5%)	7 (6.9%)	0.384
Delta, n (%)	2 (2.9%)	13 (12.7%)	0.371
Omicron, n (%)	10 (16.2%)	19 (18.6%)	0.065
**Number of infections**			
1, n (%)	46 (73.0%)	71 (69.6%)	0.000
≥2, n (%)	17 (27.0%)	31 (30.4%)	–
**Number of vaccinations**			
0–1, n (%)	0 (0.0%)	9 (8.8%)	0.816
2, n (%)	5 (7.9%)	27 (26.5%)	0.816
≥3, n (%)	57 (92.1%)	66 (64.7%)	–
**Clinical characteristics**			
ME/CFS, n (%)	0 (0.0%)	62 (61.4%)	1.783
GAD-7 score	–	5.9 ± 4.3	1.945
PHQ-9 score	–	10.4 ± 4.3	3.408
Fatigue Severity Scale	2.3 ± 1.1	5.7 ± 1.3	2.728
PCS Severity Score	0.0 ± 0.0	36.9 ± 10.6	4.931
**Laboratory parameters**			
eGFR, ml/min/1.73 m²	95.9 ± 16.3	96.4 ± 16.3	0.032
NfL, pg/ml	5.4 ± 2.2	5.9 ± 2.6	0.175
GFAP, pg/ml	59.5 ± 21.2	64.8 ± 25.4	0.224
**HGS parameters**			
Fmean, kg	29.0 ± 8.8	22.6 ± 12.5	0.591
Fatigue ratio	1.2 ± 0.1	1.3 ± 0.2	0.584
Recovery	1.0 ± 0.1	0.9 ± 0.2	0.871

#### Muscle function in the matched cohort

**Fig. 1:**
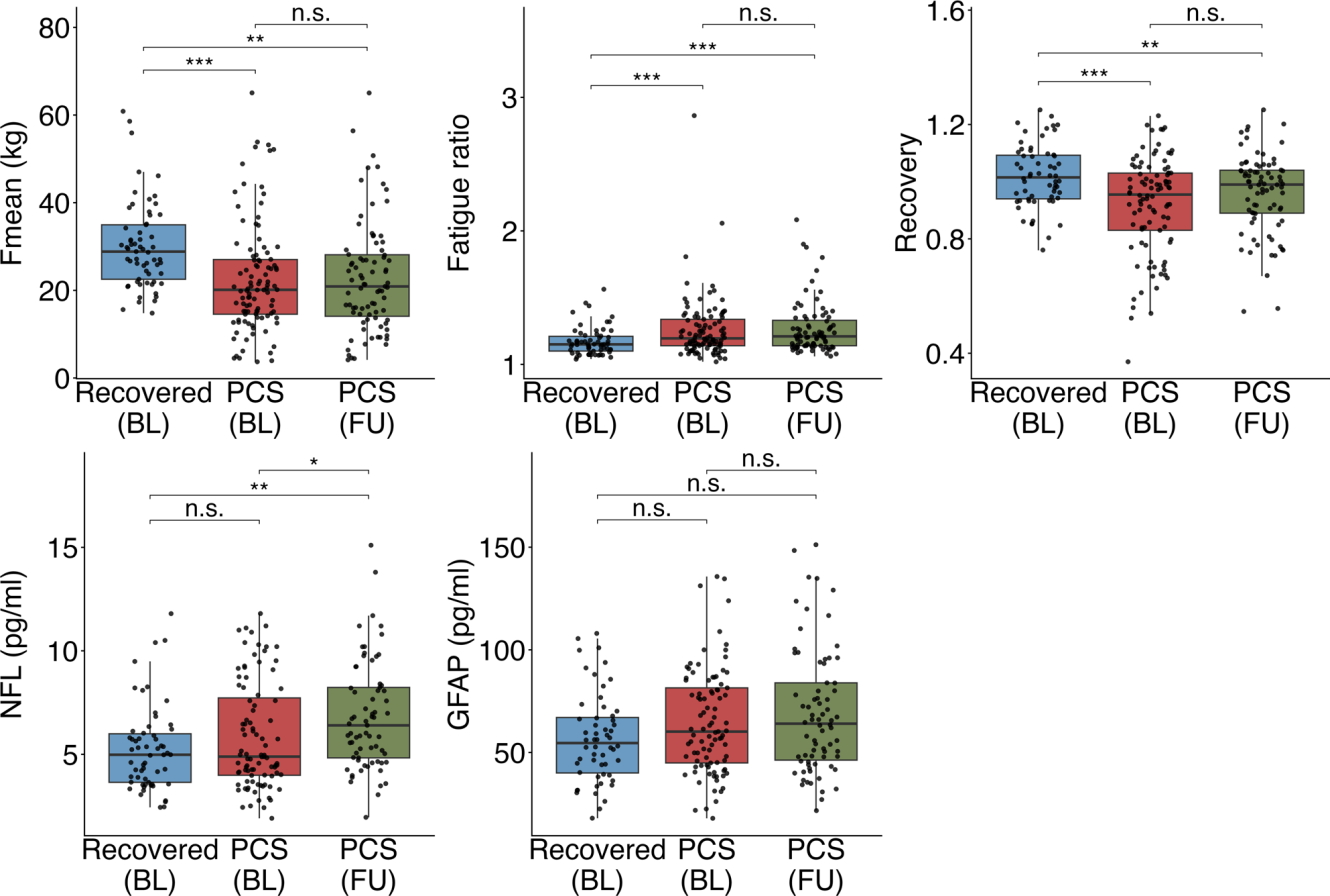
Comparison of muscle function parameters and neuroaxonal injury markers between PCS patients at baseline (BL) and follow-up (FU) and age- and sex-matched COVID-19 recovered controls assessed at BL. **a** Fmean, fatigue ratio, and recovery. **b** NfL and GFAP. Boxplots display medians and interquartile ranges; points represent individual participants. P-values are derived from weighted linear regression models using ATT propensity score weights to account for age- and sex-matching with replacement (robust HC3 standard errors). Asterisks indicate unadjusted significance levels (*p < 0.05, **p < 0.01, ***p < 0.001). Abbreviations: PCS = post-COVID syndrome; BL = baseline; FU = follow-up; NfL = neurofilament light chain; GFAP = glial fibrillary acidic protein

In the matched analyses, PCS patients exhibited persistent impairments in muscle function parameters at FU compared with recovered controls assessed at BL (Fig. 1a). Fmean was significantly lower in PCS at both BL and FU than in recovered controls (*p* < 0.001 and *p* = 0.001), with no significant difference between PCS BL and PCS FU, indicating stable impairment of muscle strength over time. Fatigue Ratio was modestly but significantly higher in PCS compared with recovered controls at BL and FU (both *p* < 0.001), while no significant change was observed within PCS over time (*p* = 0.71). Similarly, recovery was significantly lower in PCS compared with recovered controls at both BL and FU (*p* < 0.001 and *p* = 0.001), with no significant difference between PCS BL and FU (*p* = 0.087). Details of the weighted regression analysis for HGS measures are summarized in Supplementary Table 2.

#### Neuroaxonal injury markers in the matched cohort

For neuroaxonal injury markers (Fig. 1b), NfL levels did not differ between recovered controls and PCS patients at BL (*p* = 0.490), whereas significantly lower levels were observed in recovered controls compared with PCS patients at FU (ratio = 0.80, *p* = 0.004). Within the PCS cohort, NfL levels decreased over time (ratio = 0.84, *p* = 0.020). In contrast, GFAP levels did not differ significantly between groups at BL or FU (*p* = 0.363 and *p* = 0.129), and no significant change was observed within the PCS cohort over time (*p* = 0.545). Details of the weighted regression analyses for NfL and GFAP are summarized in Supplementary Table 3.

In multivariable regression models adjusting for eGFR and BMI, NfL levels were significantly higher in PCS patients at FU compared with recovered controls (β = 0.23, *p* = 0.007; Supplementary Table 4). In contrast, GFAP levels were not significantly associated with PCS status after adjustment (β = 0.10, *p* = 0.197). Lower eGFR was associated with higher GFAP levels (β = -0.009, *p* < 0.001), whereas its association with NfL did not reach statistical significance (β = -0.005, *p* = 0.083), supporting the role of renal function as a relevant confounder in biomarker analyses. In sensitivity analyses using multivariable adjustment in the full (unmatched) cohort, the association for NfL remained consistent with the matched analyses (β = 0.17, *p* = 0.006), and again no significant association was observed for GFAP after adjustment (Supplementary Table 5).

Exploratory correlation analyses within PCS patients revealed no significant associations between NfL or GFAP and HGS at BL or FU (Supplementary Fig. 2). Similarly, no significant associations were found between NfL or GFAP and either PROM at BL or FU (data not shown).

Sensitivity analyses using an alternative 1:1 nearest-neighbor matching approach without replacement yielded similar patterns for muscle function, whereas neuroaxonal injury marker findings showed variability across matching strategies, particularly for GFAP, while NfL differences were comparable (Supplementary Tables 6 and Supplementary Fig. 3).

#### Contextualization using normative reference values (full cohort)

To contextualize the clinical relevance of impaired muscle function, height-adjusted HGS values were additionally compared with published international age- and sex-specific normative reference data derived from approximately 2.4 million adults across 69 countries and regions [34], as presented in Supplementary Table 7. In the PCS cohort, 69.6% of patients were below the 10th percentile and 93.1% were below the 50th percentile. In the recovered cohort, the corresponding proportions were 38.3% and 86.8%, respectively (34.8% and 89.1% in full cohort). Values at or above the 80th percentile were uncommon in both groups (PCS: 1.0%; recovered: 2.2%).

### Muscle function in PCS (within PCS cohort analysis)

#### Longitudinal changes in muscle function and PROMs in PCS

To assess whether objective muscle impairments persist over time, longitudinal analyses were performed. As no FU HGS data were available for recovered controls, longitudinal analyses were restricted to the PCS cohort. Table 3 summarizes the longitudinal trajectory of HGS parameters and PROMs among PCS patients. Fmean and Fatigue Ratio remained essentially unchanged over six months, indicating stable physical performance. In contrast, several PROMs showed modest improvements. Muscle recovery increased significantly from 0.90 ± 0.18 to 0.96 ± 0.14 (*p* = 0.015), while Fatigue severity (FSS) and anxiety symptoms (GAD-7) decreased slightly but significantly (*p* = 0.024 and *p* = 0.007, respectively). Changes in PCS Score, PHQ-9, and C19-YRS did not reach statistical significance.

**Table 3:** Longitudinal comparison of BL and FU measures in patients with PCS. Values represent mean ± SD. Paired t-tests or Wilcoxon signed-rank tests were used depending on distribution (two-tailed)

Variable	*n*	BL (mean ± SD)	FU (mean ± SD)	Δ (mean ± SD)	*p*
Fmean	83	22.10 ± 12.35	22.75 ± 12.52	0.65 ± 11.91	0.986
Fatigue Ratio	83	1.27 ± 0.25	1.27 ± 0.20	0.00 ± 0.26	0.270
Recovery	74	0.90 ± 0.18	0.96 ± 0.14	0.05 ± 0.18	0.015
PCS Score	86	37.25 ± 10.69	35.33 ± 11.85	-1.92 ± 10.13	0.149
C19-YRS	61	32.35 ± 13.46	30.64 ± 13.86	-1.71 ± 9.96	0.185
PHQ-9	83	10.45 ± 4.43	9.75 ± 4.44	-0.70 ± 3.67	0.087
GAD-7	83	5.98 ± 4.20	5.00 ± 4.09	-0.98 ± 3.31	0.007
FSS	84	5.66 ± 1.33	5.37 ± 1.42	-0.29 ± 1.15	0.024

**Fig. 2:**
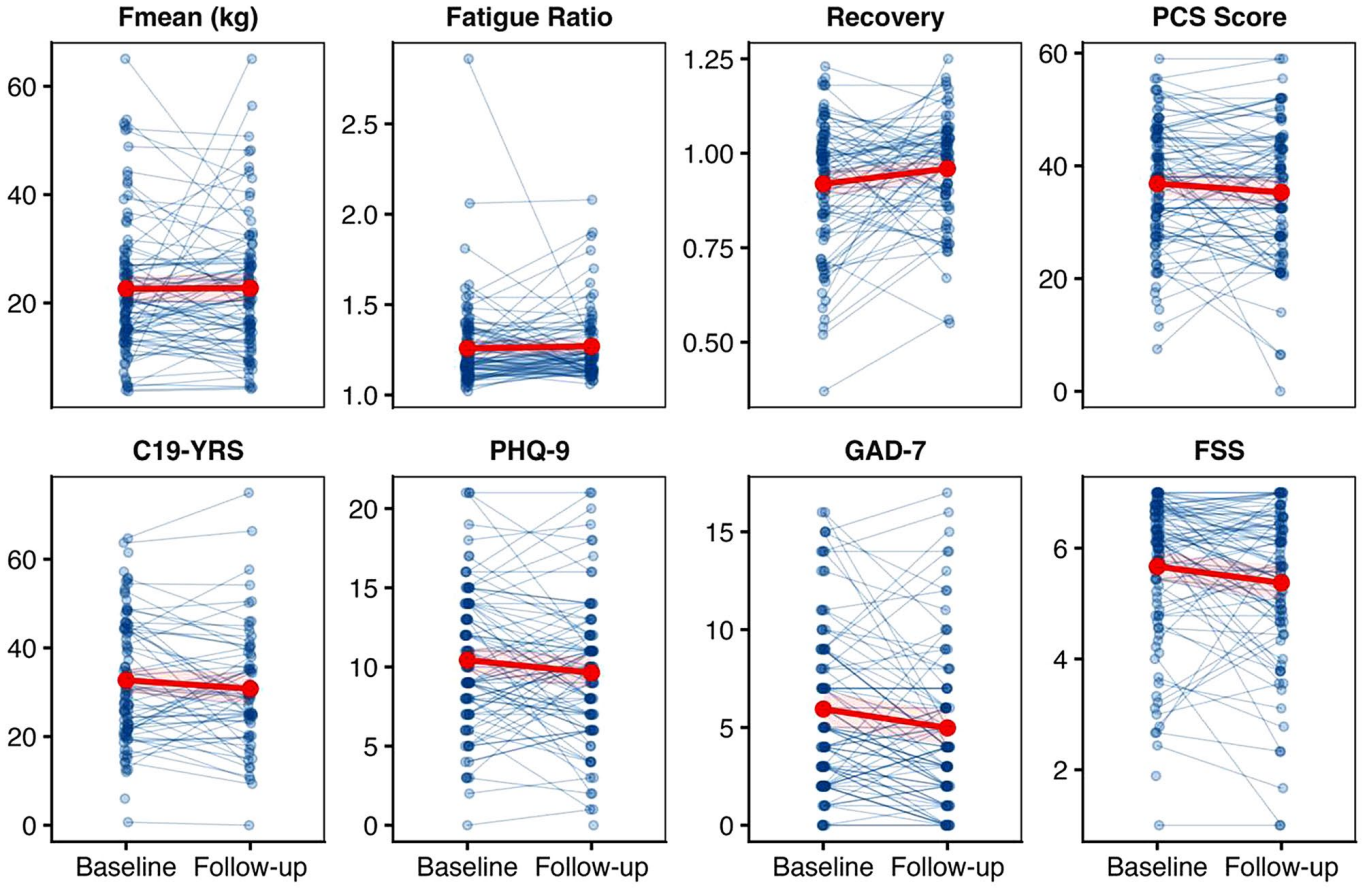
Individual longitudinal trajectories (blue lines) and group means (red lines) for HGS parameters and PROMs in PCS patients between baseline and follow-up. Each thin blue line represents an individual participant trajectory; red points and connecting lines indicate mean values with 95% confidence intervals at each timepoint. Abbreviations: PCS = post-COVID syndrome, C19-YRS = COVID-19 Yorkshire Rehabilitation Scale; PHQ-9 = Patient Health Questionnaire-9; GAD-7 = Generalized Anxiety Disorder-7; FSS = Fatigue Severity Scale

Individual longitudinal trajectories and group means of HGS measures and PROMs in the PCS cohort are shown in Fig. 2. Considerable interindividual variability was observed across all measures, with no consistent directional change over time. On a group level, mean values remained largely stable between baseline and follow-up, with only minor differences in selected parameters.

The distribution of individual change scores (Δ = FU - BL) was centered around zero across outcomes, with differing degrees of variability between parameters (Supplementary Fig. 4).

**Fig. 3:**
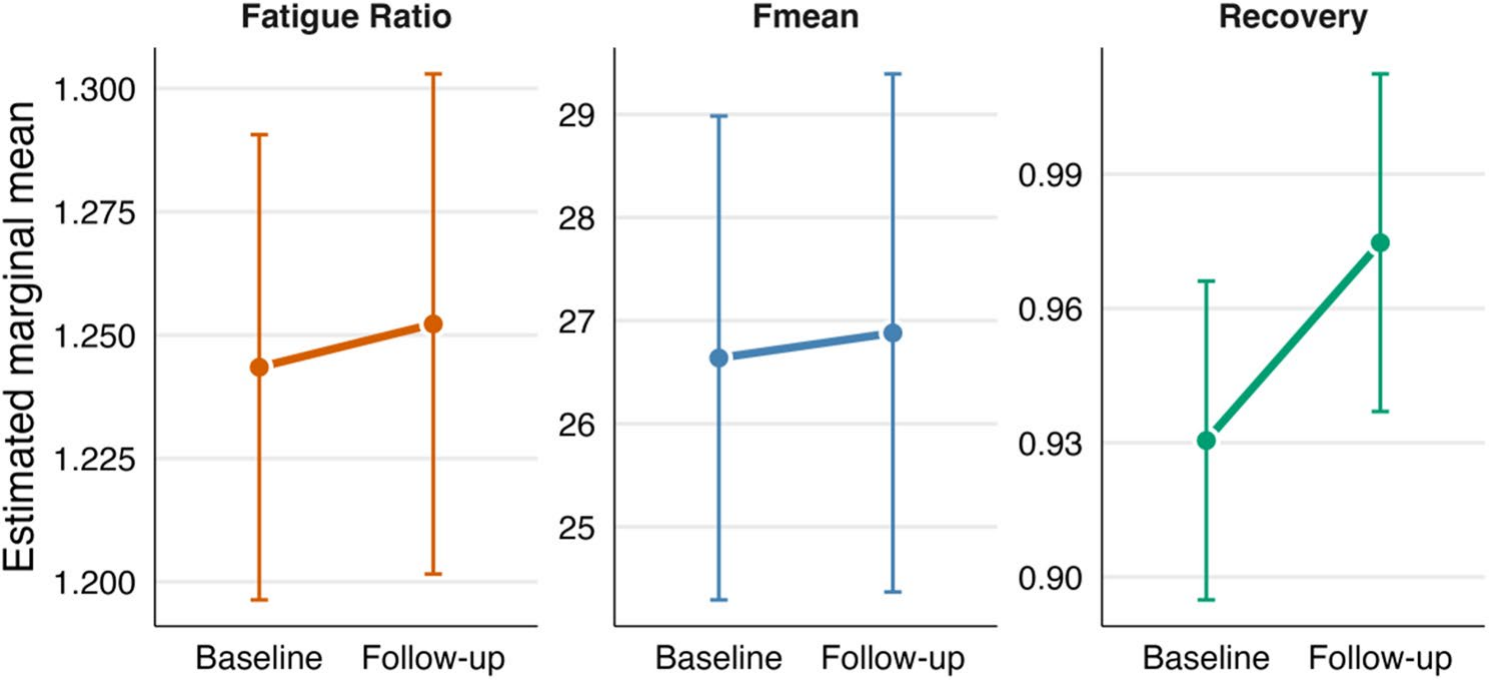
Estimated marginal means (± 95% CI) from linear mixed models adjusted for age and sex. Thick lines connect mean estimates between Baseline and Follow-up

Linear mixed models confirmed these findings, demonstrating stable Fmean and fatigability over time, with a small but significant improvement in recovery (β = 0.04, 95% CI [0.00–0.08], *p* = 0.028) (Table 4; Fig. 3).

**Table 4:** Linear mixed model results for longitudinal changes in muscle function parameters in the PCS cohort. Models included time (BL vs. FU), age, and sex as fixed effects and participant ID as a random intercept. Shown are unstandardized fixed-effect estimates (β) for the time effect with 95% confidence intervals (CIs)

Outcome	β [95% CI]	*p*
Fmean	0.24 [-2.25, 2.73]	0.847
Fatigue Ratio	0.01 [-0.04, 0.06]	0.740
Recovery	0.04 [0.00, 0.08]	0.028

#### Stability and associations of muscle function measures in PCS

Changes in HGS parameters and changes in PROMs are shown in Supplementary Table 8. No significant correlations were found for either combination.

BL muscle function measures were associated with their respective FU outcomes (Supplementary Table 9). Higher BL Fmean was strongly associated with higher FU Fmean (β = 0.55, *p* < 0.001, R² = 0.29), while greater BL fatigability was associated with lower FU Fmean (β = -11.3, *p* = 0.042, R² = 0.05). Conversely, a better BL recovery was associated with higher Fmean after six months (β = 26.9, *p* < 0.001, R² = 0.16). Similar patterns were observed for the association of FU fatigability: lower BL Fmean and recovery, and higher initial fatigability, each were associated with worse FU Fatigue Ratios. FU recovery was most strongly associated with BL recovery (β = 0.29, *p* = 0.002, R² = 0.13), suggesting temporal stability of this measure.

### Comparison of muscle function between PCS patients and recovered controls

After adjustment for age and sex, patients with PCS at FU exhibited significantly lower HGS compared with recovered controls at BL (β = -6.63, 95% CI -9.64 to -3.61, *p* < 0.001, Table 5). In addition, the fatigue ratio was significantly elevated in PCS patients (β = 0.10, 95% CI 0.05 to 0.15, *p* < 0.001). Muscle recovery was also significantly reduced in PCS patients compared with controls at BL after adjustment (β = -0.05, 95% CI -0.09 to -0.01, *p* = 0.008).

**Table 5:** Cross-sectional comparison of muscle function in patients with PCS at FU and recovered controls at BL. Values are mean ± SD. Group differences were assessed using multivariable linear regression models including group (PCS vs. control), age, and sex as covariates. Adjusted effect estimates (β), 95% confidence intervals (CI), and p-values are reported

Outcome	PCS (FU) mean ± SD	Controls (BL) mean ± SD	Adjusted β (95% CI)	Adjusted *p*
Fmean (kg)	22.75 ± 12.52	29.86 ± 10.43	-6.63 (-9.64 to -3.61)	**< 0.001**
Fatigue ratio	1.27 ± 0.20	1.19 ± 0.11	0.10 (0.05 to 0.15)	**< 0.001**
Recovery	0.96 ± 0.14	1.00 ± 0.11	-0.05 (-0.09 to -0.01)	**0.008**

### Symptom correlations and clinical associations

To evaluate the clinical relevance of these objective muscle impairments, associations with PROMs were examined.

#### Cross-sectional correlations between muscle function and PROMs in PCS

**Fig. 4:**
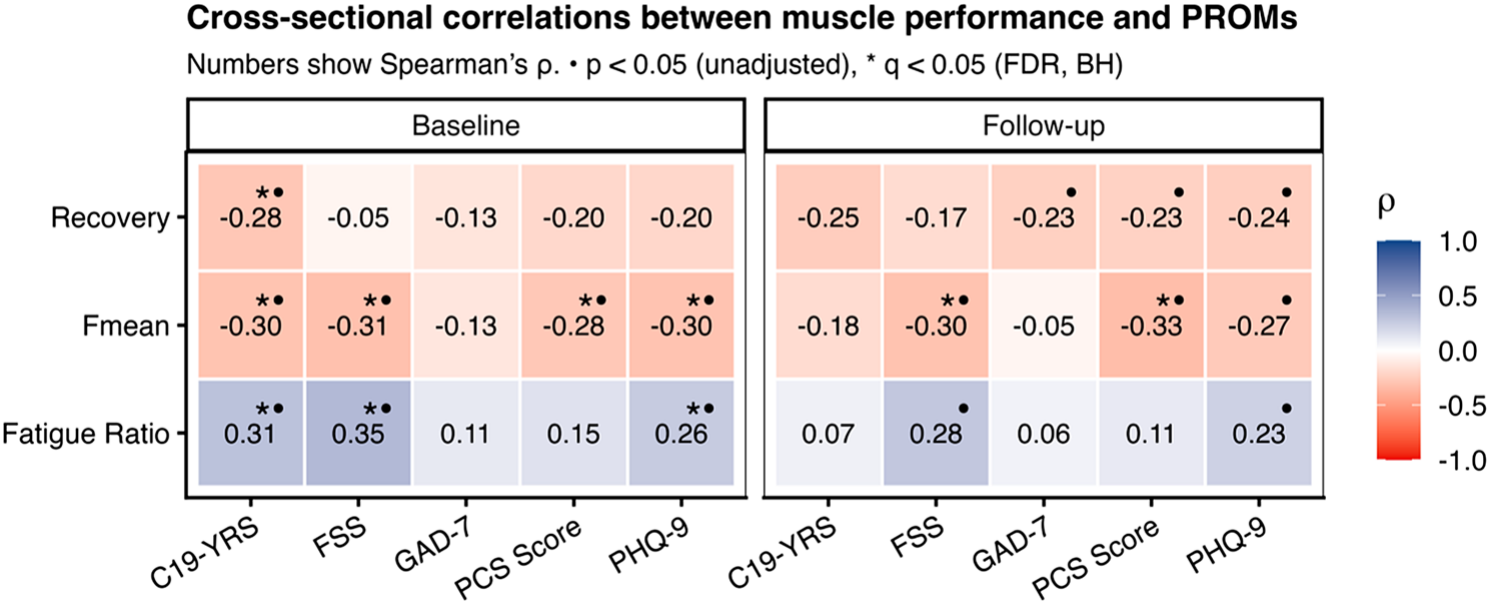
Cross-sectional correlations between muscle function parameters and patient-reported outcome measures (PROMs) in patients with PCS. Heatmaps display Spearman correlation coefficients (ρ) between objective muscle function measures (Fmean, Fatigue Ratio, Recovery) and PROMs at Baseline (left) and Follow-up (right). Numbers indicate ρ values. Dots (•) denote nominal significance (*p* < 0.05, two-sided Spearman test), and asterisks (*) denote FDR-corrected significance (q < 0.05, Benjamini-Hochberg). Color intensity reflects the direction and magnitude of correlations. Abbreviations: C19-YRS = COVID-19 Yorkshire Rehabilitation Scale; PHQ-9 = Patient Health Questionnaire-9; GAD-7 = Generalized Anxiety Disorder-7; FSS = Fatigue Severity Scale

Changes over time were assessed only within the PCS cohort, as no longitudinal data was available for the recovered cohort. At BL, Fmean was inversely correlated with fatigue severity (FSS), depressive symptoms (PHQ-9), overall PCS symptom burden, and C19-YRS scores (Spearman ρ = -0.28 to -0.31), with all associations remaining statistically significant after false discovery rate correction (Fig. 4; Supplementary Table 10). Higher muscular fatigability, quantified by the Fatigue Ratio, was positively associated with fatigue severity and C19-YRS scores (ρ = 0.31 to 0.35; q ≤ 0.011). Associations between recovery and PROMS were weaker and reached statistical significance after multiple-testing correction only for C19-YRS at BL.

At FU, correlations showed a similar direction and magnitude. Lower Fmean remained associated with greater fatigue severity (FSS; ρ = -0.30; q = 0.047) and higher overall PCS symptom burden (PCS Score; ρ = -0.33; q = 0.039). By contrast, associations between Fatigue Ratio and PROMs were attenuated and did not remain statistically significant after false discovery rate adjustment. Nominal correlations between recovery and depressive or anxiety symptoms were observed but did not remain significant after correction for multiple testing.

#### Associations between muscle function and symptom persistence in PCS

In univariable analyses, lower BL Fmean and higher fatigability were associated with greater symptom severity at FU across multiple PROMs (Supplementary Table 11). BL recovery showed inverse associations with depressive symptoms and overall symptom burden. These analyses were intended to describe unadjusted associations and informed subsequent multivariable models.

**Fig. 5:**
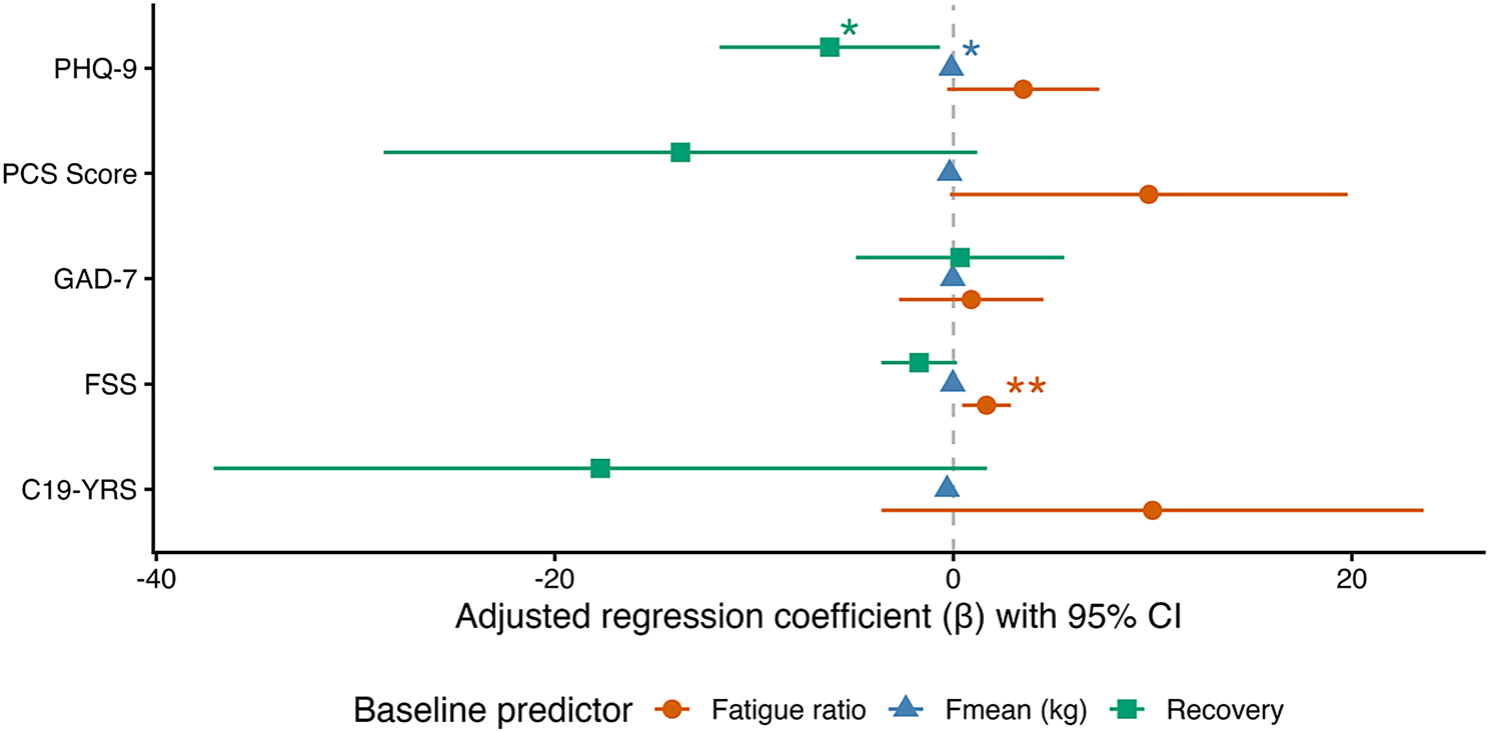
Forest plot of adjusted regression coefficients (β) with 95% confidence intervals from multivariable linear models. Points represent point estimates and horizontal lines indicate 95% confidence intervals. Colors denote baseline muscle function parameters (fatigue ratio, Fmean, recovery). Models were adjusted for age, sex, and the corresponding baseline PROM. Asterisks indicate significance levels (*p < 0.05, **p < 0.01). Abbreviations: C19-YRS = COVID-19 Yorkshire Rehabilitation Scale; PHQ-9 = Patient Health Questionnaire-9; GAD-7 = Generalized Anxiety Disorder-7; FSS = Fatigue Severity Scale

In multivariable models adjusted for age, sex, and BL symptom levels, lower BL Fmean showed a trend toward higher Fatigue Severity (FSS; β = -0.03, 95% CI [-0.06 to 0.00], *p* = 0.052) and was significantly associated with higher depressive symptom scores (PHQ-9; β = -0.11, 95% CI [-0.20 to -0.02], *p* = 0.022) at FU (Supplementary Tables 12 and Fig. 5). A higher Fatigue Ratio was associated with greater FSS (β = 1.66 [0.44 to 2.88], *p* = 0.008) and showed a trend for higher overall PCS symptom burden (*p* = 0.054). Higher recovery was associated with fewer depressive symptoms ((PHQ-9; β = -6.21, 95% CI [-11.7 to -0.68], *p* = 0.028) and showed trends for lower fatigue, PCS, and C19-YRS scores (all *p* < 0.08). No significant associations were observed for anxiety (GAD-7; all *p* > 0.49).

Interaction analyses showed no significant moderation by CFS status for any association between BL muscle function and FU PROMs (Supplementary Table 13).

## Discussion

### Principal findings

This prospective longitudinal study shows persistent impairments in muscle performance in a well-defined PCS cohort followed over six months. These findings are primarily based on propensity score-matched analyses, which were applied to reduce confounding by age and sex between groups. Our key findings demonstrate that: (1) PCS patients exhibited significantly lower Fmean, elevated muscular fatigability (Fatigue Ratio), and reduced muscle recovery compared with matched COVID-19 recovered controls at both BL and FU. Importantly, reduced HGS was evident not only relative to recovered controls but also compared with large international normative datasets; (2) these objective muscle impairments remained largely stable over the six-month observation period, with only modest improvement in recovery capacity; (3) BL muscle function parameters, particularly lower Fmean and higher Fatigue Ratio, were associated with greater symptom burden at FU, particularly depressive symptoms and fatigue, while several associations for overall PCS severity showed similar trends; and (4) circulating neuroaxonal injury markers showed inconsistent and weak associations with muscle performance, which were sensitive to adjustment for known confounders. Together, these findings support persistent reductions in HGS as a feature of chronic PCS. Baseline muscle function was associated with FU symptom burden.

### Comparison with literature

#### Persistent impairment of muscle performance in PCS

Our observation of markedly reduced HGS in PCS patients compared with recovered controls aligns with emerging evidence documenting muscle weakness as a cardinal feature of PCS [11–13]. The magnitude of impairment observed in our cohort aligns with findings by Amaral et al. [11], who reported associations between low HGS and adverse functional outcomes in affected individuals. Extending these largely cross-sectional observations, our longitudinal data demonstrate that objective muscle impairments persist over a six-month period, suggesting limited spontaneous recovery in a well-defined PCS population. However, this interpretation should be made with caution, as participants lost to FU were less severely affected at baseline, which may have led to an overrepresentation of more symptomatic individuals at FU.

By applying a multidimensional HGS protocol assessing strength (Fmean), fatigability (Fatigue Ratio), and muscle recovery, our study provides a more nuanced characterization of muscle function than maximal force measurements alone. Elevated fatigability in PCS patients indicates an accelerated decline in force generation during repeated contractions, a pattern previously described in ME/CFS using comparable protocols [14]. Prior studies have linked such fatigability metrics to post-exertional biochemical changes and mitochondrial abnormalities in post-COVID and related conditions [14, 42], supporting their biological relevance. Notably, recovery capacity showed modest improvement over time despite stable strength and fatigability, suggesting that distinct components of impaired muscle performance may follow different temporal trajectories. Importantly, longitudinal conclusions are limited to the PCS cohort, as repeated HGS assessments were not available for recovered controls, which served only as a cross-sectional reference group. As a result, it cannot be fully determined whether the observed differences reflect PCS-specific trajectories or broader post-infectious changes over time.

#### Distinction between chronic PCS and post-acute recovery

A key strength of this study is its focus on a rigorously defined chronic PCS cohort, in contrast to much of the existing literature that examined patients during early post-acute recovery. Many prior investigations assessed patients within the first three months after infection, often without standardized diagnostic criteria, complicating differentiation between delayed recovery and persistent PCS-related impairment [19–21, 43]. In contrast, participants in our cohort fulfilled WHO case definitions for PCS, with symptoms persisting beyond three months post-infection.

The use of propensity score-matched COVID-19 recovered controls further enabled isolation of PCS-specific impairments from general post-infectious effects. Persistent differences in muscle function between PCS patients and recovered controls at both BL and FU suggest that the observed deficits are unlikely to reflect normal convalescence and instead characterize a chronic PCS phenotype. This distinction is clinically relevant when interpreting persistent functional limitations in affected patients.

### Mechanisms

#### Potential mechanisms underlying persistent impairment in muscle performance

The mechanisms underlying persistent reductions of muscle performance in PCS remain incompletely understood and are likely multifactorial. Recent mechanistic studies demonstrated altered skeletal muscle metabolism, mitochondrial abnormalities, impaired oxidative phosphorylation, and a shift toward more fatigable glycolytic muscle fibers in PCS patients, particularly following exertion-induced symptom exacerbation [10]. These alterations may limit energy availability during repeated contractions and thereby contribute to increased muscular fatigability and impaired recovery in PCS. In parallel, endothelial dysfunction and impaired microvascular regulation may further reduce oxygen extraction and skeletal muscle perfusion during exertion [26]. Consistent with this concept, cardiopulmonary exercise testing studies in PCS have demonstrated impaired exercise capacity, abnormal oxygen extraction, and post-exertional symptom exacerbation, supporting the presence of persistent exertional intolerance beyond simple deconditioning [44, 45]. Neuroimmune dysregulation and autonomic dysfunction may further contribute to altered neuromuscular activation, impaired recovery kinetics, and exercise intolerance [46]. The relative stability of HGS impairments over time in our cohort may therefore reflect persistent dysregulation across interconnected metabolic, vascular, and neuroimmune pathways rather than isolated structural muscle injury. In this context, persistently impaired HGS in PCS may represent one manifestation of a broader multisystem disorder characterized by immune, vascular, and autonomic dysregulation [4, 26].

#### Prognostic relevance of baseline muscle function

A key finding of this study is that BL muscle function parameters were associated with symptom burden and functional outcomes at FU. Lower BL Fmean and higher Fatigue Ratio were associated with greater fatigue severity and depressive symptoms, while associations with overall PCS severity showed similar trends, independent of age and sex. These findings indicate that objective muscle function at initial assessment may have prognostic relevance in chronic PCS.

These associations are consistent with prior reports showing that reduced HGS early after COVID-19 is related to worse functional outcomes over time [15]. Our study extends these observations by applying a multidimensional HGS protocol capturing fatigability and recovery, and by demonstrating such associations in a rigorously defined chronic PCS cohort. The absence of significant associations between changes in HGS parameters and changes in PROMs may reflect the relative stability of both objective muscle function and symptom burden over the FU period, rather than a lack of relationship between these domains.

No significant interaction was observed between BL muscle function and comorbid ME/CFS status in relation to FU outcomes. While this finding should be interpreted cautiously given limited statistical power for interaction analyses, it suggests that the observed associations may not be restricted to specific PCS subgroups.

Although universally accepted PCS-specific thresholds for clinically meaningful HGS impairment are currently lacking, the observed between-group differences in Fmean were substantial and remained stable over time. Moreover, lower baseline Fmean and higher fatigability were consistently associated with greater symptom burden at FU, suggesting a potential clinical relevance. However, effect sizes were small and the magnitude of observed changes in PROMs and recovery was clustered around zero at the individual level, limiting their clinical interpretability. In addition, minimal clinically important differences (MCIDs) are not well established for repeated HGS-derived parameters in post-viral syndromes or for several of the PROMs used in this context, further limiting the ability to interpret the clinical significance of observed changes.

#### Neuroaxonal injury markers and impaired muscle performance

Interpretation of neuroaxonal injury biomarkers in this study requires substantial caution because circulating NfL and GFAP are influenced by several non-neurological factors, including age, renal function, BMI, and analytical strategy. In our analyses, the observed associations were modest and not fully consistent across biomarkers or modelling approaches. While NfL showed differences in some comparisons, these findings were sensitive to cohort definition, matching strategy, and adjustment approach. Additional exploratory analyses using alternative matching strategies yielded less consistent results, further limiting the robustness and interpretability of these findings. Taken together, these results do not allow firm conclusions regarding the presence, extent, or temporal dynamics of neuroaxonal injury in PCS and should be regarded as exploratory.

For GFAP, no robust association with PCS status was observed after adjustment, further limiting the strength of any pathophysiological interpretation. The fact that both eGFR and BMI showed associations with biomarker concentrations in some models underscores that peripheral biomarker levels may reflect a mixture of disease-related and non-disease-related influences. Taken together, these results should be regarded as exploratory and hypothesis-generating, particularly given the potential for residual confounding. At present, they do not provide a sufficient basis for specific conclusions about ongoing neuroaxonal or astroglial injury in PCS.

Importantly, circulating NfL and GFAP are established but non-specific markers of neuroaxonal and astroglial injury that are elevated across a broad spectrum of neurological disorders, including multiple sclerosis, traumatic brain injury, and neurodegenerative diseases [47]. Compared with these conditions, the magnitude of biomarker elevation observed in PCS appears relatively modest, arguing against severe ongoing neurodegeneration. Longitudinal studies of COVID-19-associated neuroaxonal injury markers have reported heterogeneous trajectories over time. Elevated NfL concentrations have primarily been observed in severe acute COVID-19 and critical illness, often declining during recovery phases [48, 49]. By contrast, evidence for persistently elevated biomarkers in chronic PCS remains inconsistent and appears strongly dependent on cohort characteristics, neurological symptom burden, and analytical methodology. In line with this heterogeneity, we observed no consistent associations between circulating neuroaxonal injury markers and objective muscle function measures in our cohort. The absence of consistent associations between NfL/GFAP levels and HGS parameters (Supplementary Fig. 2) may indicate that persistently impaired muscle performance in PCS is not primarily driven by ongoing large-fiber neuroaxonal injury or astroglial damage detectable in peripheral blood. Instead, HGS abnormalities may reflect functional disturbances at the metabolic, mitochondrial, vascular, autonomic, or neuromuscular junction level that are insufficient to induce measurable increases in circulating neuroaxonal injury markers. In this context, retinal microvascular alterations, as investigated in the overarching “All Eyes on PCS” project, may serve as a proxy of systemic microvascular dysfunction. Given that the retinal microvasculature is widely regarded as a non-invasive window into systemic vascular health [50], the observed HGS impairments are therefore unlikely to represent isolated skeletal muscle deficits but may instead reflect a broader systemic pathophysiology. Importantly, circulating NfL and GFAP primarily reflect structural neuroaxonal or astroglial injury and may therefore not capture predominantly functional or metabolic alterations. As a result, the absence of strong biomarker associations should not be interpreted as evidence against neurobiological contributions to impaired muscle function in PCS.

### Clinical implications

A key implication of this study is that objective HGS parameters provide a robust functional correlate of PROMs in PCS, both at BL and at FU. The limited improvement in PROMs over six months suggests that symptom burden may remain relatively stable in a substantial proportion of patients with PCS. The consistent associations between measures of muscle strength, fatigability, and recovery and validated PROMs across multiple symptom domains support the potential use of HGS as an objective complement to subjective symptom assessment. The observed pattern of exertional intolerance and persistent fatigability also resembles findings reported in other post-viral syndromes, particularly ME/CFS [14, 51].

Importantly, the persistence of these associations over time indicates that HGS parameters continue to reflect symptom burden beyond the initial presentation, reinforcing their relevance for longitudinal characterization of PCS cohorts. In this context, objective muscle function measures may help to contextualize and substantiate patient-reported fatigue, weakness, and functional impairment.

From a methodological perspective, the integration of standardized HGS assessment alongside PROMs may improve the phenotypic characterization and comparability of PCS cohorts in both clinical and research settings. While the present findings do not support therapeutic recommendations, they suggest that objective functional measures such as HGS may represent a valuable component of comprehensive PCS assessment. Because HGS assessment is inexpensive, rapid, and widely implementable, it may represent a practical objective adjunct to PROM-based PCS assessment in both outpatient and research settings.

### Limitations

Several limitations of this study should be acknowledged. First, the observational design precludes causal inference regarding the relationship between impaired muscle performance and symptom persistence in PCS. Although BL muscle function parameters were associated with FU symptom burden, reverse causation cannot be excluded.

Second, comparisons with published normative HGS values should be interpreted with caution, as our handgrip metric was based on the mean of 10 repeated contractions, whereas the reference percentiles were harmonized to three trials. These analyses therefore provide an external benchmark, but not a protocol-identical comparison. Furthermore, HGS was not normalized to measures of body composition such as lean mass or fat-free mass. As a result, reduced HGS may reflect a combination of decreased muscle mass, physical inactivity, and deconditioning rather than intrinsic muscle dysfunction per se.

Third, the FU period captures medium-term trajectories but may be insufficient to characterize longer-term disease courses. It remains unclear whether objective muscle impairments and patient-reported symptoms eventually resolve, remain stable, or progress over longer time horizons.

Fourth, the moderate sample size, particularly after accounting for loss to FU, limits statistical power to detect small effect sizes and to perform detailed subgroup analyses. Loss to FU represents a potential source of selection bias. Although not statistically significant, non-completers showed a tendency toward lower baseline symptom burden and slightly more favorable muscle function measures. This pattern suggests a possible, albeit non-significant, enrichment of more severely affected individuals at FU. This may result in an overrepresentation of more severely affected individuals at FU, potentially leading to an overestimation of symptom persistence and an underestimation of improvement over time. Moreover, the recruitment strategy resulted in a cohort with relatively high symptom burden, which may limit generalizability. In addition, residual confounding cannot be excluded. Factors such as physical activity, deconditioning, pain, sleep disturbance, depressive symptoms, overlap with ME/CFS, severity of the acute infection, vaccination status, and time since infection may have influenced HGS, circulating biomarker levels, and symptom burden. Although most of these variables were partially captured, the study was not designed to comprehensively adjust for all potential confounders. Therefore, the observed associations should be interpreted with caution.

Fifth, biomarker findings should be interpreted as exploratory, as effect sizes were moderate and sensitive to analytical strategy. The study was not specifically powered for biomarker analyses, and statistical power was further reduced in the primary matched analyses due to propensity score weighting. Although 63 unique control individuals contributed to the matched sample, weighting resulted in a reduced effective sample size, which may have contributed to the instability of some findings. In additional sensitivity analyses using an alternative 1:1 nearest-neighbor matching approach without replacement, similar effect patterns were observed for muscle function. NfL and GFAP findings were generally consistent, although some variability, particularly for GFAP, remained across matching strategies. Circulating NfL and GFAP are influenced by non-neurological factors such as age, renal function, and BMI, which may attenuate disease-specific signals despite adjustment. Moreover, these markers may be insensitive to subtle or non-cytodestructive neurological alterations in PCS. Differences across studies may reflect heterogeneity in PCS definitions, disease duration, symptom severity, and methodological approaches.

Finally, despite the application of rigorous diagnostic criteria, PCS remains a heterogeneous condition. The present findings may therefore not be uniformly applicable across all PCS phenotypes, underscoring the need for larger longitudinal studies with finer phenotypic stratification.

### Conclusion

In conclusion, persistent impairment in objective muscle function represents a stable and clinically relevant feature of chronic PCS. Extending prior predominantly cross-sectional observations, our longitudinal study demonstrates that multidimensional HGS-derived parameters are associated with symptom burden and longitudinal outcomes, supporting their potential value as objective markers of functional impairment. In contrast, circulating neuroaxonal injury markers did not show a consistent and robust relationship with muscle performance, suggesting that the underlying mechanisms may be only partially overlapping or difficult to disentangle. Together, these findings support the integration of objective muscle function assessment into longitudinal PCS characterization.

### Summary table

**Table:** 

**What is known about this subject:**
Post-COVID syndrome is defined by persistent symptoms after SARS-CoV-2 infection and is diagnosed clinically.
Objective measures to complement symptom-based assessment in PCS are limited.
Handgrip strength is an established, non-invasive measure of global muscle function.
What this paper adds:
Handgrip strength parameters capture persistent physical impairment in a well-defined PCS cohort.
Objective muscle function is associated with patient-reported outcomes at baseline and follow-up.
Neuroaxonal injury markers showed no consistent elevation in PCS and no consistent association with muscle function.

## Supplementary Information

Below is the link to the electronic supplementary material.

Supplementary Material 1

## Data Availability

The data underlying this manuscript will be shared upon reasonable request to the corresponding author. All shared data will be fully anonymized/de-identified to ensure participant privacy in accordance with institutional and GDPR regulations.
