# Supplementary material for: Persistent impairments in muscle function and symptom burden in post-COVID syndrome: a prospective longitudinal study
Source: J Transl Med. 2026 Jul 7;24:871. doi: 10.1186/s12967-026-08579-z (PMC13348007; doi:10.1186/s12967-026-08579-z)
Supplement: Supplementary file 1 — Supplementary Material 1 [file 12967_2026_8579_MOESM1_ESM.docx]

Supplementary Material

# Persistent Impairments in Muscle Function and Symptom Burden in Post-COVID Syndrome: A Prospective Longitudinal Study

Michael Wunderle^1*^, Andrea Ribeiro^1^, Isabelle Lethen^1^, Christoph Schmaderer^1,2,3^, Timon Wallraven^1^

^1^TUM University Clinic, Klinikum rechts der Isar, Department of Nephrology, Technical University of Munich, 81675 Munich, Germany

^2^German Centre for Infection Research (DZIF), Munich Partner Site, Munich, Germany

^3^Site Lead, VADYS-ME Research Program, TUM University Clinic, Munich

# Characteristics of Follow-Up Data

Baseline comparisons between PCS participants with and without FU data are shown in Supplementary Table 1. No statistically significant differences were observed across demographic, clinical, muscle function, or biomarker variables (all p > 0.05). Although not statistically significant, participants lost to FU showed a tendency toward lower baseline symptom burden and slightly more favorable muscle function measures.

| **Variable** | **Completers n = 87** | **Non-completers n = 15** | **p-value** |
| --- | --- | --- | --- |
| Age (years) | 41.95 ± 11.41 | 41.33 ± 12.85 | 0.730 |
| Male sex, n (%) | 22 (25.3%) | 3 (20.0%) | 1.000 |
| ME/CFS, n (%) | 54 (62.1%) | 8 (57.1%) | 0.772 |
| C19-YRS | 33.21 ± 13.99 | 27.39 ± 15.66 | 0.325 |
| PCS Score | 37.25 ± 10.63 | 34.53 ± 10.24 | 0.424 |
| GAD-7 | 6.00 ± 4.18 | 5.57 ± 5.26 | 0.451 |
| PHQ-9 | 10.40 ± 4.37 | 10.64 ± 4.22 | 0.608 |
| FSS | 5.67 ± 1.31 | 5.63 ± 1.47 | 0.953 |
| Fmean (kg) | 22.37 ± 12.56 | 24.08 ± 12.64 | 0.571 |
| Fatigue ratio | 1.27 ± 0.24 | 1.20 ± 0.11 | 0.271 |
| Recovery | 0.91 ± 0.17 | 0.99 ± 0.16 | 0.081 |
| NfL (pg/ml) | 5.72 ± 2.52 | 6.54 ± 2.81 | 0.317 |
| GFAP (pg/ml) | 64.45 ± 26.73 | 66.51 ± 17.83 | 0.555 |

**Supplementary Table 1:** Baseline comparison of participants with and without FU data. Values are presented as mean ± standard deviation or percentages as appropriate. Group differences were assessed using Wilcoxon rank-sum tests for continuous variables and Fisher’s exact test for categorical variables.

# Matched Analysis

Covariate balance was substantially improved after propensity score matching. Prior to matching, large imbalances were observed for age and the propensity score, whereas sex was already well balanced. After matching, standardized mean differences for all matching variables were reduced to values close to zero and remained well below the predefined threshold of 0.1, indicating excellent covariate balance between PCS patients and COVID-19 recovered controls (Supplementary Figure 1).


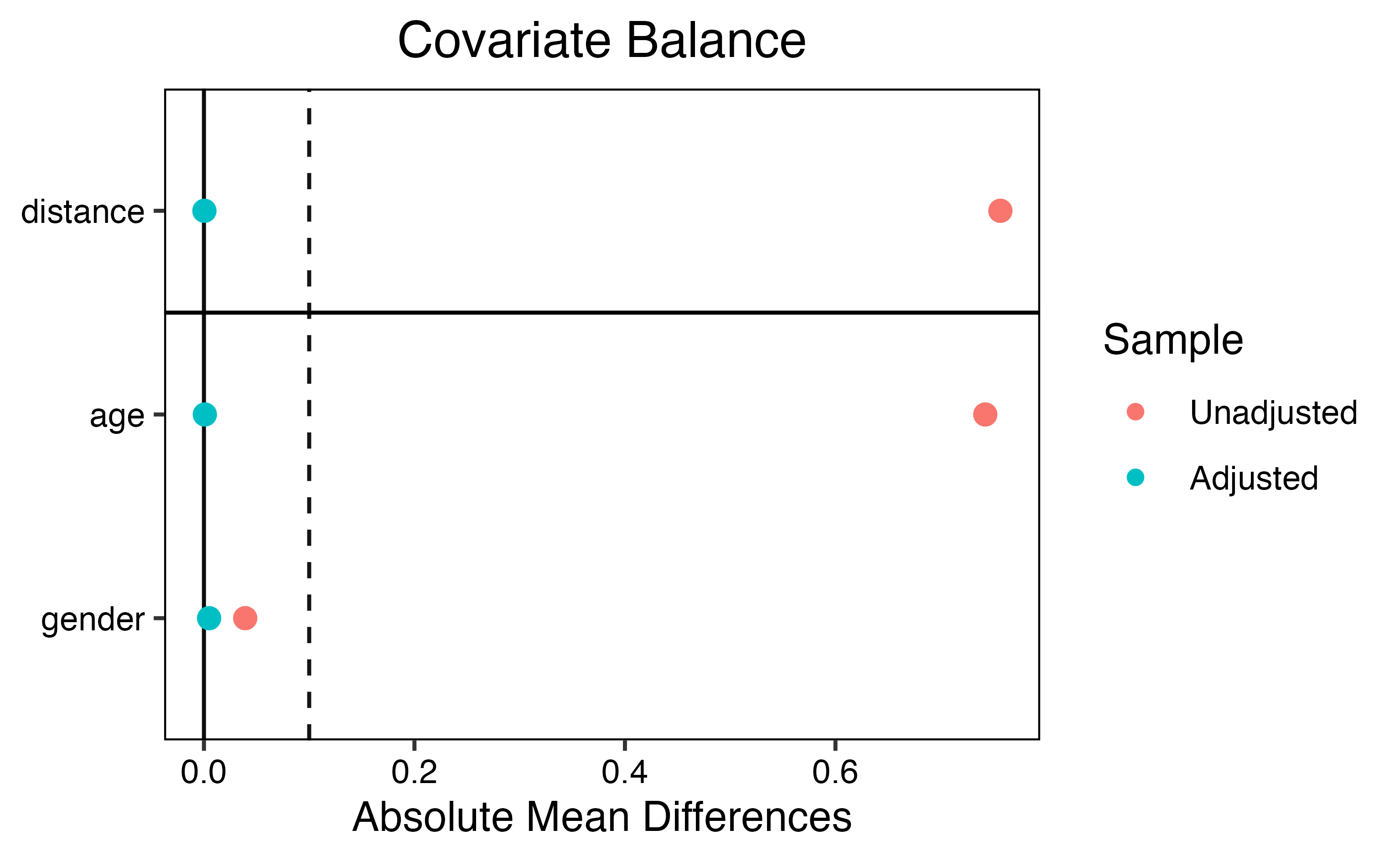


**Supplementary Figure 1:** Supplementary Figure 1. Love plot showing standardized mean differences (SMDs) before (unadjusted) and after (adjusted) weighted propensity score matching. Points represent absolute SMDs for each covariate. The dashed vertical line indicates the threshold for adequate balance (SMD < 0.1). Colors denote unadjusted and adjusted samples.

Weighted regression analyses corroborated the descriptive findings by formally quantifying group differences after accounting for the propensity score-based matching (Supplementary Table 2). For muscle function parameters, Fmean was significantly lower in PCS compared with recovered controls at both BL and FU, while Fatigue Ratio was significantly higher. Recovery was modestly but significantly lower in PCS relative to controls at both timepoints. No significant changes were observed between PCS BL and PCS FU for any muscle function parameter in the weighted models, consistent with the largely stable trajectories observed in the longitudinal analyses.

| Outcome | Contrast | β (SE) | p-value |
| --- | --- | --- | --- |
| Fmean | Recovered (BL) vs PCS (BL) | 6.39 (1.81) | **<0.001** |
| Fmean | Recovered (BL) vs PCS (FU) | 6.27 (1.91) | **0.001** |
| Fmean | PCS (BL) vs PCS (FU) | -0.12 (1.86) | 0.947 |
| Fatigue ratio | Recovered (BL) vs PCS (BL) | -0.10 (0.027) | **<0.001** |
| Fatigue ratio | Recovered (BL) vs PCS (FU) | -0.11 (0.026) | **<0.001** |
| Fatigue ratio | PCS (BL) vs PCS (FU) | -0.01 (0.032) | 0.714 |
| Recovery | Recovered (BL) vs PCS (BL) | 0.13 (0.028) | **<0.001** |
| Recovery | Recovered (BL) vs PCS (FU) | 0.09 (0.027) | **0.001** |
| Recovery | PCS (BL) vs PCS (FU) | -0.04 (0.024) | 0.087 |

**Supplementary Table 2:** Weighted linear regression contrasts for Fmean, Fatigue Ratio, and Recovery comparing PCS at BL and FU with COVID-19–recovered controls assessed at BL. Models used ATT propensity score weights with robust HC3 standard errors. β denotes unstandardized regression coefficients.

For neuroaxonal injury markers in matched analysis, NfL and GFAP differed significantly between PCS and recovered controls at both BL and FU, whereas no significant differences were observed between PCS BL and PCS FU, indicating temporal stability of these markers within PCS over the FU period (Supplementary Table 3).

| Outcome | Contrast | β (SE) | Ratio | p-value |
| --- | --- | --- | --- | --- |
| NfL | Recovered (BL) vs PCS (BL) | -0.05 (0.08) | 0.95 | 0.490 |
| NfL | Recovered (BL) vs PCS (FU) | -0.23 (0.08) | 0.80 | **0.004** |
| NfL | PCS (BL) vs PCS (FU) | -0.17 | 0.84 | **0.020** |
| GFAP | Recovered (BL) vs PCS (BL) | -0.07 (0.08) | 0.93 | 0.363 |
| GFAP | Recovered (BL) vs PCS (FU) | -0.13 (0.08) | 0.88 | 0.129 |
| GFAP | PCS (BL) vs PCS (FU) | -0.04 | 0.96 | 0.545 |

**Supplementary Table 3:** Weighted linear regression contrasts for NfL and GFAP comparing PCS at BL and FU with COVID-19 recovered controls assessed at BL. Models were fitted using ATT propensity score weights with robust HC3 standard errors. Estimates are shown on the log scale; ratios represent exponentiated coefficients.

In multivariable weighted regression models adjusting for eGFR and BMI in the matched cohort, PCS status was significantly associated with higher NfL levels compared with COVID-19–recovered controls (Supplementary Table 4). In contrast, no significant association between PCS status and GFAP levels was observed after adjustment. eGFR showed a significant inverse association with GFAP but not with NfL, whereas BMI was not associated with NfL and showed a modest inverse association with GFAP.

| **Outcome** | **Predictor** | **β (SE)** | **GMR (95% CI)** | **p-value** |
| --- | --- | --- | --- | --- |
| NfL (log) | PCS vs recovered | 0.23 (0.08) | 1.26 (1.07–1.47) | **0.007** |
|  | eGFR (per ml/min/1.73 m²) | -0.005 (0.003) | 1.00 (0.99–1.00) | 0.083 |
|  | BMI (kg/m²) | -0.008 (0.009) | 0.99 (0.98–1.01) | 0.375 |
| GFAP (log) | PCS vs recovered | 0.10 (0.08) | 1.11 (0.95–1.30) | 0.197 |
|  | eGFR (per ml/min/1.73 m²) | -0.009 (0.003) | 0.99 (0.99–1.00) | **<0.001** |
|  | BMI (kg/m²) | -0.023 (0.010) | 0.98 (0.96–1.00) | **0.032** |

**Supplementary Table 4:** Multivariable weighted linear regression models for neuroaxonal injury markers at FU. For PCS patients, FU values were analyzed, whereas BL values were used for COVID-19–recovered controls. Models were fitted on the log scale and adjusted for eGFR and BMI using ATT propensity score weights. Regression coefficients (β) with standard errors (SE), p-values, and geometric mean ratios (GMR) with 95% confidence intervals (CI) are reported.

Multivariable linear regression models of the full (unmatched) cohort were fitted on the log scale to compare FU values in patients with PCS with baseline values in COVID-19–recovered controls (Supplementary Table 5). Models were adjusted for age, sex, estimated glomerular filtration rate (eGFR) and BMI. PCS status remained significantly associated with higher NfL levels, whereas no significant association was observed for GFAP. Age remained independently associated with both biomarkers, while BMI showed a modest inverse association with GFAP but not with NfL.

| **Outcome** | **Predictor** | **β (SE)** | **GMR (95% CI)** | **p-value** |
| --- | --- | --- | --- | --- |
| **NfL (log)** | PCS vs recovered | 0.17 (0.06) | 1.19 (1.05–1.34) | **0.006** |
|  | Age (per year) | 0.011 (0.003) | 1.01 (1.00–1.02) | **<0.001** |
|  | Male sex | -0.10 (0.06) | 0.91 (0.80–1.02) | 0.115 |
|  | eGFR (per ml/min/1.73 m²) | -0.001 (0.002) | 1.00 (0.99–1.00) | 0.563 |
|  | BMI (kg/m²) | -0.008 (0.006) | 0.99 (0.98–1.00) | 0.200 |
|  | Model fit |  | Adjusted R² = 0.24 |  |
| **GFAP (log)** | PCS vs recovered | 0.11 (0.07) | 1.12 (0.97–1.29) | 0.113 |
|  | Age (per year) | 0.010 (0.004) | 1.01 (1.00–1.02) | **0.005** |
|  | Male sex | -0.13 (0.07) | 0.88 (0.76–1.01) | 0.070 |
|  | eGFR (per ml/min/1.73 m²) | -0.004 (0.002) | 1.00 (0.99–1.00) | 0.138 |
|  | BMI (kg/m²) | -0.017 (0.007) | 0.98 (0.97–1.00) | **0.016** |
|  | Model fit |  | Adjusted R² = 0.21 |  |

**Supplementary Table 5:** Multivariable linear regression models fitted on the log scale comparing biomarker levels between PCS patients at FU and COVID-19–recovered controls at BL in the full cohort. Models were adjusted for age, sex, estimated glomerular filtration rate (eGFR) and BMI. Regression coefficients (β) with standard errors (SE), p-values, and geometric mean ratios (GMR) with 95% confidence intervals (CI) are reported. Sex was coded as male versus female (reference).

Exploratory correlation analyses revealed no significant associations between NfL and GFAP and HGS parameters, including Fmean, Fatigue Ratio, and Recovery, at either BL or FU (Supplementary Figure 2). Correlation coefficients were consistently small and did not reach statistical significance, indicating that circulating neuroaxonal injury markers were not directly related to peripheral muscle function in this PCS cohort.


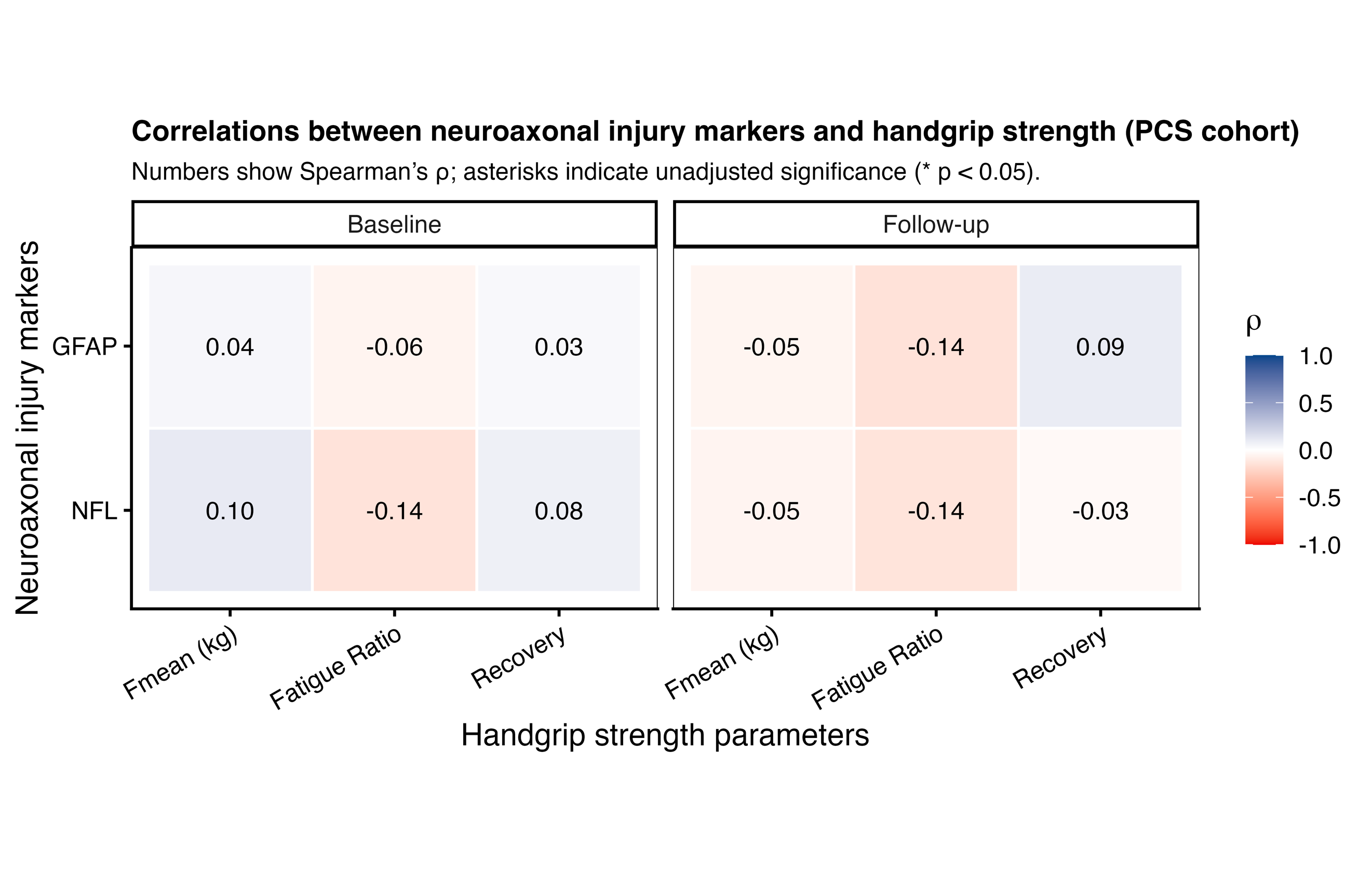


**Supplementary Figure 2:** Heatmaps showing Spearman correlation coefficients (ρ) between neuroaxonal injury markers (NfL, GFAP) and handgrip strength parameters (Fmean, fatigue ratio, recovery) at Baseline and Follow-up. Values indicate ρ coefficients; asterisks (*) denote nominal significance (p < 0.05). Color intensity reflects the direction and magnitude of correlations. Abbreviations: NfL = neurofilament light chain; GFAP = glial fibrillary acidic protein.

# Alternative Matching Strategy

Baseline characteristics in the alternative 1:1 propensity score-matched cohort without replacement are shown in Supplementary Table 6. These data were already published [1]. Matching resulted in comparable distributions of key covariates, including age, sex, and BMI, between PCS patients and recovered controls. Differences in muscle function parameters were consistent with the primary matched analysis, with lower Fmean, higher fatigue ratio, and reduced recovery observed in PCS patients. For neuroaxonal injury markers, effect estimates showed some variability across matching approaches, with GFAP levels differing significantly between groups in the 1:1 matched cohort, whereas findings were less consistent in the primary weighted analysis.

| Variable | COVID-19 recovered  (n = 71) | PCS patients  (n = 71) | p-value |
| --- | --- | --- | --- |
| Age (years), median (IQR) | 34.0 (28.0–45.5) | 36.0 (28.5–50.0) | 0.37 |
| Female sex n (%) | 50 (70.4) | 55 (77.5) | 0.44 |
| BMI (kg/m²), median (IQR) | 23.0 (21.4–24.9) | 23.6 (20.6–25.8) | 0.99 |
| Laboratory markers | | | |
| NfL (pg/ml), median (IQR) | 5.3 (4.0–6.1) | 5.0 (4.1–8.1) | 0.48 |
| GFAP (pg/ml), median (IQR) | 55.1 (37.4–67.3) | 61.4 (45.5–85.7) | 0.016 |
| HGS parameters | | | |
| Fmean (kg) | 29.0 (23.7 - 35.0) | 19.9 (13.9 - 25.5) | < 0.001 |
| Fatigue Ratio | 1.1 (1.1 - 1.2) | 1.2 (1.1 - 1.3) | 0.006 |
| Recovery | 1.0 (0.9 - 1.1) | 0.9 (0.8 - 1.0) | < 0.001 |

**Supplementary Table 6:** Baseline characteristics of PCS patients and COVID-19 recovered controls using an alternative 1:1 nearest neighbor matching approach without replacement. Continuous variables are presented as median (interquartile range) or mean ± SD, and categorical variables as n (%). Group comparisons were performed using t-test or Wilcoxon rank-sum test for continuous variables and χ² or Fisher’s exact test for categorical variables, as appropriate.

Supplementary Figure 3 shows the results of the alternative 1:1 matched analysis. Differences in muscle function parameters were comparable to the primary matched analysis, with lower Fmean, higher fatigue ratio, and reduced recovery observed in PCS patients at both BL and FU.

For neuroaxonal injury markers, NfL levels differed between recovered controls at BL and PCS patients at FU, whereas no consistent differences were observed at baseline. GFAP levels were higher in PCS patients compared with recovered controls, with a clearer separation between groups than in the primary weighted analysis.

**Supplementary Figure 3:** Comparison of muscle function parameters and neuroaxonal injury markers between patients with PCS at BL and FU and 1:1 age- and sex-matched COVID-19 recovered controls assessed at BL. **a** Fmean, fatigue ratio, and recovery. **b** NfL and GFAP. Boxplots show baseline values in recovered controls and baseline and follow-up values in PCS patients after 1:1 nearest-neighbor matching without replacement. Group comparisons were performed using unweighted linear models with heteroscedasticity-robust HC3 standard errors, followed by pairwise estimated marginal mean contrasts without multiplicity adjustment. Asterisks indicate unadjusted significance levels (*p < 0.05, **p < 0.01, ***p < 0.001). Abbreviations: PCS = post-COVID syndrome; BL = baseline; FU = follow-up; NfL = neurofilament light chain; GFAP = glial fibrillary acidic protein.

# Comparison with Normative Data

Compared with recovered controls, PCS patients showed a markedly greater proportion of individuals with reduced Fmean relative to international normative values [2] (Supplementary Table 7). Specifically, nearly 70% of PCS patients fell below the 10th percentile, compared with approximately 35% of recovered controls, indicating substantial impairment. Although a high proportion of both groups fell below the median of the reference population, PCS patients consistently exhibited lower adjusted Fmean across all percentile thresholds.

| **Normative threshold** | **PCS** | **Recovered controls** |
| --- | --- | --- |
| Mean adjusted Fmean (kg/m²) | 7.0 | 9.9 |
| <10th percentile, n (%) | 71 (69.6) | 32 (34.8) |
| <20th percentile, n (%) | 79 (77.5) | 49 (53.3) |
| <50th percentile, n (%) | 95 (93.1) | 82 (89.1) |
| ≥80th percentile, n (%) | 1 (1.0) | 2 (2.2) |

**Supplementary Table 7:** Distribution of height-adjusted Fmean relative to international normative reference values. Adjusted Fmean was defined as Fmean normalized to height (kg/m²) and compared with age- and sex-specific percentile distributions derived from a large international reference population. Values are presented as the proportion of participants falling below selected percentile thresholds (<10th, <20th, <50th) and above the ≥80th percentile.

# Longitudinal Changes in HGS and PROMs in PCS

The distribution of individual change scores (Δ = FU - BL) was approximately symmetric across outcomes, with values centered around zero (Supplementary Figure 4).


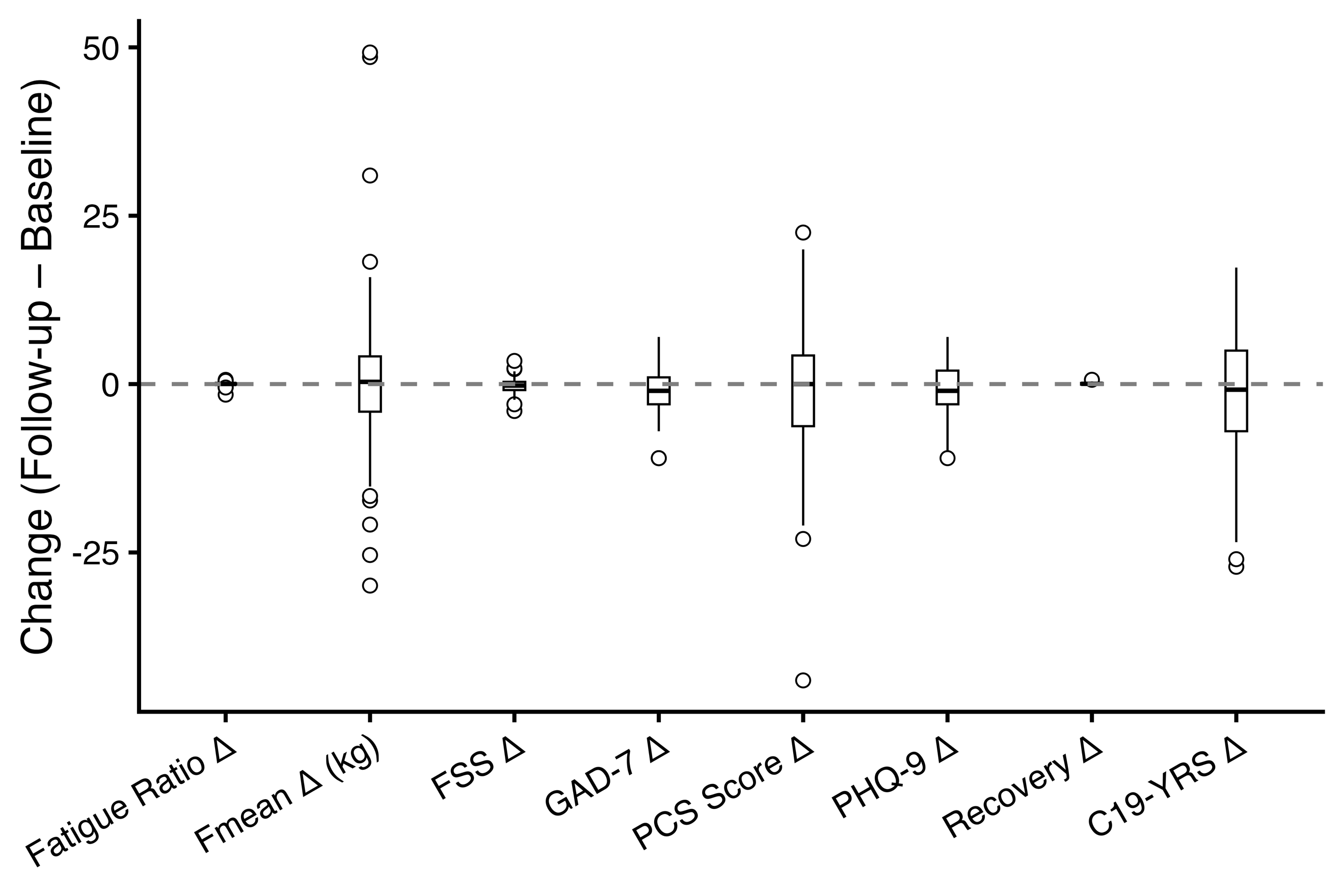


**Supplementary Figure 4:** Distribution of individual change scores (Δ = FU - BL) across all outcomes in patients with PCS. Boxplots display the median (horizontal line), interquartile range (box), and distribution of Δ values. The dashed line at 0 indicates no change. Abbreviations: PCS = post-COVID syndrome, C19-YRS = COVID-19 Yorkshire Rehabilitation Scale; PHQ-9 = Patient Health Questionnaire-9; GAD-7 = Generalized Anxiety Disorder-7; FSS = Fatigue Severity Scale.

Changes in HGS parameters and changes in PROMs are shown in Supplementary Table 8. No significant correlations were found for either combination.

| ΔObjective | ΔPROM | n | ρ (Spearman) | p-value | p (FDR) |
| --- | --- | --- | --- | --- | --- |
| ΔFatigue Ratio | ΔFSS | 80 | 0.12 | 0.289 | 0.856 |
| ΔFmean | ΔFSS | 80 | -0.02 | 0.861 | 0.940 |
| ΔRecovery | ΔFSS | 72 | 0.20 | 0.099 | 0.777 |
| ΔFatigue Ratio | ΔGAD-7 | 79 | -0.07 | 0.550 | 0.856 |
| ΔFmean | ΔGAD-7 | 79 | 0.07 | 0.520 | 0.856 |
| ΔRecovery | ΔGAD-7 | 71 | -0.17 | 0.155 | 0.777 |
| ΔFatigue Ratio | ΔPCS | 82 | 0.05 | 0.625 | 0.856 |
| ΔFmean | ΔPCS | 82 | -0.05 | 0.673 | 0.856 |
| ΔRecovery | ΔPCS | 74 | -0.05 | 0.685 | 0.856 |
| ΔFatigue Ratio | ΔPHQ-9 | 79 | 0.05 | 0.678 | 0.856 |
| ΔFmean | ΔPHQ-9 | 79 | 0.06 | 0.602 | 0.856 |
| ΔRecovery | ΔPHQ-9 | 71 | -0.02 | 0.877 | 0.940 |
| ΔFatigue Ratio | ΔYRS | 58 | -0.09 | 0.482 | 0.856 |
| ΔFmean | ΔYRS | 58 | 0.00 | 0.995 | 0.995 |
| ΔRecovery | ΔYRS | 55 | -0.21 | 0.132 | 0.777 |

**Supplementary Table 8:** Pairwise Spearman correlations between changes (Δ = FU - BL) in HGS measures and changes in PROMs. Correlations were computed with pairwise complete observations; p-values adjusted using the Benjamini-Hochberg procedure.

Univariate linear regression models (Supplementary Table 9) were fitted to examine whether BL muscle function parameters were associated with their corresponding FU parameters. Fmean at BL was significantly associated with FU handgrip strength (β = 0.55, p < 0.001), while higher BL recovery was associated with higher FU Fmean (β = 26.9, p < 0.001). BL fatigue ratio was inversely associated with FU Fmean (β = –11.3, p = 0.042) and FU fatigue ratio (β = 0.28, p = 0.001). Similarly, BL recovery was associated with FU fatigue ratio (β = –0.41, p = 0.001).

| FU outcome | BL predictor | β (Estimate) | SE | p*-*value | R² |
| --- | --- | --- | --- | --- | --- |
| Fmean | Fmean | 0.55 | 0.09 | **<0.001** | 0.29 |
| Fmean | Fatigue Ratio | -11.3 | 5.48 | **0.042** | 0.05 |
| Fmean | Recovery | 26.9 | 7.11 | **<0.001** | 0.16 |
| Fatigue Ratio | Fmean | -0.005 | <0.01 | **0.005** | 0.09 |
| Fatigue Ratio | Fatigue Ratio | 0.28 | 0.08 | **0.001** | 0.12 |
| Fatigue Ratio | Recovery | -0.41 | 0.12 | 0.001 | 0.13 |
| Recovery | Fmean | 0.002 | <0.01 | 0.161 | 0.03 |
| Recovery | Fatigue Ratio | -0.019 | 0.06 | 0.757 | 0.00 |
| Recovery | Recovery | 0.29 | 0.09 | **0.002** | 0.13 |

**Supplementary Table 9:** Univariable linear regression analyses assessing associations between BL function measures and FU muscle function in patients with PCS. Each model includes one predictor variable. β = unstandardized regression coefficient; SE = standard error; R² = explained variance.

**Supplementary Table 10** summarizes the cross-sectional Spearman correlations (ρ) between HGS parameters and PROMs in the PCS cohort at BL and at six-month FU. Across both time points, lower HGS and higher muscular fatigability were consistently associated with greater symptom severity as measured by the Fatigue Severity Scale (FSS), the C19-YRS Symptom Severity score, and the PHQ-9. Correlations with other PROMs were weaker and did not reach significance after correction for multiple testing (FDR).

| Objective variable | PROM | n | ρ (Spearman) | p-value | p (FDR) |
| --- | --- | --- | --- | --- | --- |
| BL | | | | | |
| Fmean | PCS Score | 102 | -0.28 | **0.005** | **0.013** |
| Fatigue Ratio | PCS Score | 102 | 0.15 | 0.145 | 0.197 |
| Recovery | PCS Score | 96 | -0.20 | 0.057 | 0.085 |
| Fmean | C19-YRS | 92 | -0.30 | **0.004** | **0.011** |
| Fatigue Ratio | C19-YRS | 92 | 0.31 | **0.003** | **0.011** |
| Recovery | C19-YRS | 86 | -0.28 | **0.009** | **0.017** |
| Fmean | GAD-7 | 100 | -0.13 | 0.213 | 0.246 |
| Fatigue Ratio | GAD-7 | 100 | 0.11 | 0.288 | 0.309 |
| Recovery | GAD-7 | 94 | -0.13 | 0.200 | 0.246 |
| Fmean | PHQ-9 | 100 | -0.30 | **0.002** | **0.011** |
| Fatigue Ratio | PHQ-9 | 100 | 0.26 | **0.009** | **0.017** |
| Recovery | PHQ-9 | 94 | -0.20 | 0.053 | 0.085 |
| Fmean | FSS | 101 | -0.31 | **0.001** | **0.011** |
| Fatigue Ratio | FSS | 101 | 0.35 | **<0.001** | **0.005** |
| Recovery | FSS | 95 | -0.05 | 0.610 | 0.610 |
| FU | | | | | |
| Fmean | PCS Score | 82 | -0.33 | **0.003** | **0.039** |
| Fatigue Ratio | PCS Score | 82 | 0.11 | 0.344 | 0.430 |
| Recovery | PCS Score | 79 | -0.23 | **0.045** | 0.085 |
| Fmean | C19-YRS | 60 | -0.18 | 0.177 | 0.241 |
| Fatigue Ratio | C19-YRS | 60 | 0.07 | 0.584 | 0.631 |
| Recovery | C19-YRS | 59 | -0.25 | 0.055 | 0.092 |
| Fmean | GAD-7 | 80 | -0.05 | 0.669 | 0.669 |
| Fatigue Ratio | GAD-7 | 80 | 0.06 | 0.588 | 0.631 |
| Recovery | GAD-7 | 77 | -0.23 | **0.042** | 0.085 |
| Fmean | PHQ-9 | 80 | -0.27 | **0.017** | 0.063 |
| Fatigue Ratio | PHQ-9 | 80 | 0.23 | **0.042** | 0.085 |
| Recovery | PHQ-9 | 77 | -0.24 | **0.035** | 0.085 |
| Fmean | FSS | 80 | -0.30 | **0.006** | **0.047** |
| Fatigue Ratio | FSS | 80 | 0.28 | **0.013** | 0.063 |
| Recovery | FSS | 77 | -0.18 | 0.129 | 0.194 |

**Supplementary Table 10:** Spearman correlations (ρ) between HGS parameters and PROMs at BL and six-month FU in the PCS cohort. Negative ρ values indicate that lower physical performance is associated with higher symptom severity. p-values are corrected for multiple testing using the Benjamini–Hochberg false-discovery rate (FDR).

Univariate linear regression models (Supplementary Table 11) examined BL Fmean, fatigue ratio, and recovery as predictors of FU PROMs (FSS, PHQ-9, GAD-7, PCS Score, and C19-YRS). Lower BL Fmean was associated with higher fatigue (p = 0.016), depressive symptoms (p = 0.002), PCS scores (p = 0.004), and C19-YRS severity (p = 0.015). Higher fatigue ratio was associated with greater fatigue (p = 0.006), depression (p = 0.035), and PCS symptom burden (p= 0.039). Higher recovery predicted fewer depressive symptoms (p = 0.015) and lower PCS scores (p = 0.043), with trends for fatigue and C19-YRS (both p < 0.06). No significant associations were found for anxiety (all p > 0.17).

| FU PROM | BL predictor | β (95% CI) | p-value | R² |
| --- | --- | --- | --- | --- |
| FSS (Fatigue) | Handgrip strength | -0.03 (-0.05, -0.01) | **0.016** | 0.07 |
|  | Fatigue Ratio | 1.69 (0.51, 2.88) | **0.006** | 0.09 |
|  | Recovery | -1.79 (-3.62, 0.04) | 0.059 | 0.05 |
| PHQ-9 (Depression) | Handgrip strength | -0.12 (-0.20, -0.05) | **0.002** | 0.11 |
|  | Fatigue Ratio | 4.23 (0.36, 8.10) | **0.035** | 0.05 |
|  | Recovery | -6.97 (-12.5, -1.49) | **0.015** | 0.07 |
| GAD-7 (Anxiety) | Handgrip strength | -0.05 (-0.12, 0.02) | 0.178 | 0.02 |
|  | Fatigue Ratio | 1.31 (-2.24, 4.87) | 0.471 | 0.01 |
|  | Recovery | -0.07 (-5.17, 5.02) | 0.977 | 0.00 |
| PCS Score | Handgrip strength | -0.29 (-0.48, -0.10) | **0.004** | 0.10 |
|  | Fatigue Ratio | 10.8 (0.72, 20.8) | **0.039** | 0.05 |
|  | Recovery | -15.5 (-30.4, -0.7) | **0.043** | 0.05 |
| C19-YRS | Handgrip strength | -0.34 (-0.60, -0.07) | **0.015** | 0.09 |
|  | Fatigue Ratio | 11.6 (-1.6, 24.8) | 0.091 | 0.05 |
|  | Recovery | -18.7 (-37.4, 0.10) | 0.056 | 0.06 |

**Supplementary Table 11:** Prediction of FU PROMs by BL muscle function parameters among patients with PCS. Values represent unstandardized regression coefficients (β) with 95% confidence intervals and p-values. R² denotes the proportion of variance explained by each model.

Multivariable linear regression models adjusted for age, sex, and the corresponding BL PROM were used to assess prognostic associations between BL HGS parameters and FU outcomes (Supplementary Table 12). Lower BL Fmean was associated with higher fatigue (p = 0.052) and higher depressive symptoms (p = 0.022), with trends for higher PCS and C19-YRS scores (p = 0.12 and p = 0.058, respectively). Higher fatigue ratio was associated with greater fatigue (p = 0.008) and showed trends for higher PCS symptom burden (p= 0.054) and depression (p = 0.071). Higher recovery was associated with fewer depressive symptoms (p = 0.028) and showed trends for lower fatigue, PCS, and C19-YRS scores (all p < 0.08). No significant associations were observed for anxiety (all p > 0.49).

| Outcome | Predictor | β [95% CI] | p-value | R² |
| --- | --- | --- | --- | --- |
| FSS | Fmean | -0.03 [-0.06 – 0.00] | 0.052 | 0.07 |
|  | Fatigue Ratio | 1.66 [0.44 – 2.88] | **0.008** | 0.11 |
|  | Recovery | -1.72 [-3.61 – 0.17] | 0.074 | 0.06 |
| PHQ-9 | Fmean | -0.11 [-0.20 – -0.02] | **0.022** | 0.17 |
|  | Fatigue Ratio | 3.50 [-0.31 – 7.32] | 0.071 | 0.14 |
|  | Recovery | -6.21 [-11.7 – -0.68] | **0.028** | 0.13 |
| GAD-7 | Fmean | -0.03 [-0.12 – 0.06] | 0.492 | 0.05 |
|  | Fatigue Ratio | 0.90 [-2.72 – 4.51] | 0.624 | 0.04 |
|  | Recovery | 0.34 [-4.89 – 5.56] | 0.899 | 0.02 |
| PCS Score | Fmean | -0.19 [-0.44 – 0.05] | 0.123 | 0.12 |
|  | Fatigue Ratio | 9.80 [-0.17 – 19.8] | 0.054 | 0.13 |
|  | Recovery | -13.7 [-28.6 – 1.19] | 0.071 | 0.12 |
| C19-YRS | Fmean | -0.32 [-0.65 – 0.01] | 0.058 | 0.11 |
|  | Fatigue Ratio | 9.99 [-3.61 – 23.6] | 0.147 | 0.09 |
|  | Recovery | -17.7 [-37.1 – 1.68] | 0.073 | 0.08 |

**Supplementary Table 12:** Linear regression models adjusted for age, sex, and the corresponding BL PROM. Shown are unstandardized regression coefficients (β) with 95% confidence intervals (CI) and p-values.

No significant interaction effects between BL muscle function and CFS status were observed for any of the examined PROMs (Supplementary Table 13). This indicates that the associations between muscle function measures (Fmean, Fatigue Ratio, Recovery) and FU symptom scores did not differ between PCS patients with and without ME/CFS.

| Predictor (BL) | Outcome (FU) | β [95% CI] | p |
| --- | --- | --- | --- |
| **Fmean** | GAD-7 | 0.10 [-0.03, 0.22] | 0.139 |
|  | FSS | -0.03 [-0.09, 0.03] | 0.391 |
|  | PCS Score | 0.11 [-0.25, 0.47] | 0.542 |
|  | PHQ-9 | 0.03 [-0.07, 0.12] | 0.557 |
|  | C19-YRS | 0.06 [-0.32, 0.44] | 0.744 |
| **Fatigue Ratio** | PCS Score | -12.8 [-42.0, 16.3] | 0.383 |
|  | C19-YRS | -8.14 [-37.1, 20.8] | 0.575 |
|  | PHQ-9 | -4.41 [-13.8, 5.02] | 0.354 |
|  | FSS | 0.83 [-1.82, 3.49] | 0.534 |
|  | GAD-7 | -0.82 [-19.9, 18.3] | 0.932 |
| **Recovery** | PCS Score | 2.48 [-29.5, 34.5] | 0.878 |
|  | C19-YRS | 2.07 [-53.7, 57.8] | 0.941 |
|  | GAD-7 | -4.10 [-15.0, 6.85] | 0.458 |
|  | PHQ-9 | -4.97 [-17.4, 7.43] | 0.427 |
|  | FSS | -0.99 [-5.00, 3.01] | 0.622 |

**Supplementary Table 13:** Interaction effects of BL muscle function with CFS status on FU PROMs. Unstandardized regression coefficients (β) with 95% CIs from ANCOVA models adjusted for age, sex, and BL outcome.

# References

1. Wunderle, M., et al., *Serum NfL and GFAP in post-COVID syndrome: minimal evidence of CNS injury after adjusting for confounders.* Front Cell Neurosci, 2026. **20**: p. 1750121.

2. Tomkinson, G.R., et al., *International norms for adult handgrip strength: A systematic review of data on 2.4 million adults aged 20 to 100+ years from 69 countries and regions.* J Sport Health Sci, 2025. **14**: p. 101014.
